# Improved GNSS integer ambiguity resolution method based on the column oriented Cholesky decomposition

**DOI:** 10.1038/s41598-023-31635-3

**Published:** 2023-03-17

**Authors:** Yingxiang Jiao, Kezhao Li, Chendong Tian, Guoku Zhu, Zhe Yue, Keke Xu

**Affiliations:** 1 School of Surveying and Land Information Engineering, Henan Polytechnic University, Jiaozuo, 454000 China; 2 Collaborative Innovation Center of BDS Research Application, Zhengzhou, 450052 China

**Keywords:** Engineering, Aerospace engineering, Civil engineering, Electrical and electronic engineering

## Abstract

Because the traditional Cholesky decomposition algorithm still has some problems such as computational complexity and scattered structure among matrices when solving the GNSS ambiguity,  it is the key problem to further improve the computational efficiency of the least squares ambiguity reduction correlation process in the carrier phase integer ambiguity solution. But the traditional matrix decomposition calculation is more complex and time-consuming, to improve the efficiency of the matrix decomposition, in this paper, the decomposition process of traditional matrix elements is divided into two steps: multiplication update and column reduction of square root calculation. The column reduction step is used to perform square root calculation and column division calculation, while the update step is used for the update task of multiplication. Based on the above ideas, the existing Cholesky decomposition algorithm is improved, and a column oriented Cholesky (C-Cholesky) algorithm is proposed to further improve the efficiency of matrix decomposition, so as to shorten the calculation time of integer ambiguity reduction correlation. The results show that this method is effective and superior, and can improve the data processing efficiency by about 12.34% on average without changing the integer ambiguity accuracy of the traditional Cholesky algorithm.

## Introduction

Global navigation satellite system (GNSS) is an air-based radio navigation and positioning system, which can provide users with all-weather three-dimensional coordinates and time information at any place on the earth's surface or near earth space^1^. Where, the key to whether GNSS can achieve high-precision differential positioning is the rapid solution of carrier phase integerc ambiguity^2–5^. At present, a large number of scholars have also proposed many algorithms to improve the integer ambiguity resolution. Counselman and Gourevitch first proposed an integer ambiguity function (IAF) method which takes the maximum value of integer ambiguity function as the criterion and uses spatial coordinates as the search space to obtain the coordinates of the survey station^6^. Melbourne and Wübbena proposed a M–W combination of dual-frequency code phase combination, which can form the combined observation value by selecting the appropriate linear combination coefficient of the integer ambiguity, and then the combined integer ambiguity after linear conversion is gradually rounded to obtain the integer ambiguity^7,8^. The three carrier ambiguity resolution (TCAR) proposed by Forssell et al.^9^ for the European GNSS-2 program, and the cascading integer resolution (CIR) proposed by Hatch et al.^10^. Kim and Langley proposed an optimal GPS ambiguity estimation method based on the least square method. The search space was reduced by using the scaling and filtering process, achieving ultrahigh performance and computing efficiency^11^.

The least-squares ambiguity decorrelation adjustment (LAMBDA) proposed by Teunissen et al.^12–14^ is the best and most widely used to solve the integer ambiguity. The core idea of LAMBDA algorithm is to reduce the correlation of the covariance matrix to improve the subsequent search efficiency. However, a large number of matrix operations are involved in the processing of ambiguity decorrelation correlation, which greatly affects the efficiency of ambiguity resolution. Therefore, it is necessary to further study its decorrelation process to improve the efficiency of ambiguity resolution.

A large number of scholars have carried out a series of research on the basis of the reduced correlation theory of LAMBDA algorithm, and proposed a large number of improved algorithms. A joint reduced correlation algorithm based on the construction of upper and lower triangular processes, which further reduced the correlation of the covariance matrix was proposed by Liu^15^. An improved LAMBDA algorithm was proposed by Chang, which based on the principles of greedy selection and partial decorrelation, simplifying the complexity of the algorithm^16^. A fuzzy decorrelation algorithm with better performance based on the criterion of reducing the condition number of covariance matrix was presented in this paper written by Chen^17^. Wang et al. presented a constrained LAMBDA method to get fixed GPS integer ambiguity resolution, which can obtain the attitude information using the known conditions, when the sufficiency accurate floating solution and variance covariance matrix can not be provided^18^. A multi constraint LAMBDA method was proposed by Giorgi^19^. This method can improve the ability to fix the correct integer ambiguity set, using some nonlinear geometrical constraints to strengthen basic observation model. Liu and Zhang^20^ proposed a global optimization integer ambiguity algorithm based on the artificial fish swarm algorithm (AF), which can provide higher integer ambiguity solution efficiency and strong robustness. An inverse integer Cholesky decomposition algorithm was proposed to improve the efficiency of reducing correlation^21^. Liu and Huang^22^ proposed that the correlation LAMBDA process can be improved by reducing the dimension of the ambiguity covariance matrix, which overcomes the matrix ill conditioned decomposition problem that may be caused by Z-transform. Li et al.^23^ improved LAMBDA algorithm based on Tikhonov regularization principle. By performing singular value decomposition on the coefficient matrix of the double difference observation equation, the regularization matrix was selected to improve the ill condition of the normal matrix and obtain a floating-point solution with higher accuracy, and the speed and success rate of integer ambiguity solution were improved, by replacing the covariance matrix with the mean square error matrix. The influence of different ranking methods on the reduced correlation when pre-ranking the covariance matrix^24–27^. A decorrelation algorithm using upper and lower triangular Cholesky decomposition, which obtained a better reduced correlation effect and significantly improved the success rate of the solution of the transformed ambiguity vector^28^.

To sum up, the decorrelation processing efficiency in the ambiguity resolution process of GNSS will directly affect the resolution efficiency of the whole ambiguity. However, the Cholesky decomposition in the traditional algorithm still has some problems, such as complex calculation, scattered matrix structure, and so on. Therefore, this paper proposes an improved column-oriented Cholesky decomposition algorithm (C-Cholesky). C-Cholesky algorithm converts the traditional decomposition process into two steps of column vector reduction and update, and parallelizes the above two steps to improve the resolution efficiency during decomposition, and then shortens the resolution time of integer ambiguity.

## Theory and methods

### Mathematical model of LAMBDA ambiguity resolution

In GNSS high-precision carrier phase differential positioning, the double difference observation equation can be linearized into

1 $${\mathbf{y}} = {\mathbf{Aa}} + {\mathbf{Bb}} + {\mathbf{e}}.$$

In Eq. (1), $y$ is the carrier phase double difference observation value, ${\mathbf{a}}$ is the double difference ambiguity vector, ${\mathbf{b}}$ is the unknown of baseline vector after double difference,${\mathbf{A}}$ and ${\mathbf{B}}$ are designed matrices of ambiguity and baseline respectively, ${\mathbf{e}}$ is the error vector.

To solve Eq. (1), it can be transformed into a constrained least-squares problem according to the least-squares criterion. The formula is as follows

2 $$\mathop {\min }\limits_{a,b} \left\| {y - {\mathbf{A}}a - {\mathbf{B}}b} \right\|_{{Q_{y} }}^{2} \quad a \in Z^{n} ,b \in R^{n} .$$

In Eq. (2), $\left\| . \right\|_{{Q_{y} }}^{2} = \left( . \right)^{ * } {\mathbf{Q}}_{y}^{ - 1} \left( . \right)$, ${\mathbf{Q}}_{y}$ is the covariance matrix of double difference carrier phase observations.

In order to solve the least-squares problem, we first need to eliminate the constraints in Eq. (2). That is, ignore the integer property of $$a$$ and treat it as a real number to obtain the real estimate of ambiguity $$\hat{a}$$ and its corresponding variance covariance matrix, and then substitute the obtained result into Eq. (3) to solve ambiguity $$\overset{\lower0.5em\hbox{$\smash{\scriptscriptstyle\smile}$}}{a}$$.

3 $$\mathop {\min }\limits_{a} \left( {a - \hat{a}} \right)^{\rm T} {\mathbf{Q}}_{{\hat{a}}}^{{{ - }1}} \left( {\hat{a} - a} \right)\quad a \in Z^{n} .$$

Once $$\overset{\lower0.5em\hbox{$\smash{\scriptscriptstyle\smile}$}}{a}$$ is obtained, the residual $$(\hat{a} - \overset{\lower0.5em\hbox{$\smash{\scriptscriptstyle\smile}$}}{a} )$$ can be used to solve the baseline solution $$\overset{\lower0.5em\hbox{$\smash{\scriptscriptstyle\smile}$}}{b}$$.

4 $$\overset{\lower0.5em\hbox{$\smash{\scriptscriptstyle\smile}$}}{b} - \hat{b}\left| {\overset{\lower0.5em\hbox{$\smash{\scriptscriptstyle\smile}$}}{a} } \right. = \hat{b} - {\mathbf{Q}}_{{\hat{b}\hat{a}}} {\mathbf{Q}}_{{\hat{a}}}^{ - 1} \left( {\hat{a} - \overset{\lower0.5em\hbox{$\smash{\scriptscriptstyle\smile}$}}{a} } \right),$$

where $$\hat{a}$$ and $$\hat{b}$$ are usually called ambiguity floating-point solutions. In addition, $$\overset{\lower0.5em\hbox{$\smash{\scriptscriptstyle\smile}$}}{a}$$ and $$\overset{\lower0.5em\hbox{$\smash{\scriptscriptstyle\smile}$}}{b}$$ are called ambiguity fixed solutions.

In order to make the process of solving ambiguity $$\overset{\lower0.5em\hbox{$\smash{\scriptscriptstyle\smile}$}}{a}$$ more efficient, integer GAUSS transform (Z transform) can be used to reduce the correlation between ambiguity components. Transform the least-squares problem of Eq. (3) into a new least-squares problem

5 $$\mathop {\min }\limits_{z} \left( {z - \hat{z}} \right)^{\rm T} {\mathbf{Q}}_{{\hat{z}}}^{{{ - }1}} \left( {\hat{z} - z} \right)\quad {\text{z}} \in Z^{n} ,$$

6 $$z = {\mathbf{Z}}^{\rm T} {\text{a}}, \, \hat{z} = {\mathbf{Z}}^{\rm T} {\hat{\text{a}}}, \, {\mathbf{Q}}_{{\hat{z}}} = {\mathbf{Z}}^{\rm T} {\mathbf{Q}}_{a} {\mathbf{Z}}.$$

In Eqs. (5) and (6): ${\mathbf{Z}}$ matrix is a unimodular matrix, that is, the determinant of the matrix is 1 and the elements of matrix ${\mathbf{Z}}$ are integers.

### Cholesky decomposition model

According to the relevant knowledge of linear algebra, the decomposition of Hermitian matrices can be called the product of a lower triangular matrix ${\mathbf{L}}$ and the transpose of Hermitian matrices by using the Cholesky decomposition algorithm. If the column vector of the matrix ${\mathbf{L}}$ is normalized with respect to its diagonal elements, the second form of Cholesky decomposition, namely ${\mathbf{LDU}}$ decomposition, can be obtained. Where, ${\mathbf{L}}$ matrix elements and ${\mathbf{U}}$ are symmetrical and equal about the main diagonal. The covariance matrix ${\mathbf{Q}}$ can be regarded as a Hermite matrix with all real numbers, which can be decomposed by Cholesky decomposition algorithm. Because the second form of Cholesky decomposition avoids the operation on the square root, reduces the loss of calculation accuracy, and it is easy to implement in engineering. The second form of Cholesky decomposition is usually used to further process the covariance matrix in the process of reducing correlation of LAMBDA algorithm^29^.

${\mathbf{Q}}_{{{\hat{\text{a}}}}}$ and ${\mathbf{Q}}_{{\hat{z}}}$ in Eq. (6) are decomposed by the second form of Cholesky decomposition

7 $${\mathbf{Q}}_{{\hat{a}}} = {\mathbf{L}}^{\rm T} {\mathbf{DL}}, \, {\mathbf{Q}}_{{\hat{z}}} = {\mathbf{Z}}^{\rm T} {\mathbf{L}}^{\rm T} {\mathbf{DLZ}} = \overline{{\mathbf{L}}}^{\rm T} \overline{{{\mathbf{DL}}}}$$

where the ${\mathbf{L}}$ and $\overline{{\mathbf{L}}}$ are unit lower triangular matrices, ${\mathbf{D}}$ and $\overline{{\mathbf{D}}}$ are diagonal element and all diagonal elements are greater than 0. The construction equation of ${\mathbf{Z}}$ matrix is

8 $${\mathbf{Z}} = {\mathbf{I}} - \eta e_{i} e_{j}^{\rm T} ,$$

where the ${\mathbf{I}}$ is the n-dimensional identity matrix, $\eta$ is the rounding of the elements of row $i$ and column $j$ of matrix ${\mathbf{L}}$, $e_{i}$ and $e_{j}$ are the unit vector coordinate.

When decomposing the covariance matrix ${\mathbf{Q}}$, the calculation formulas of each element are as follow

9 $$\begin{aligned} & l_{ij} { = }\left( {q_{ij} - \sum\limits_{k = 1}^{j - 1} {l_{ik} u_{kj} d_{kk} } } \right){\text{ /d}}_{jj} \, \\ & \quad i = 2,3, \ldots ,n;j = 1,2, \ldots ,i - 1, \\ \end{aligned}$$

10 $$\begin{aligned} & d_{ii} = q_{ii} - \sum\limits_{k = 1}^{i - 1} {l_{ik} u_{ki} d_{kk} } \\ & \quad i = 1,2, \ldots ,n, \\ \end{aligned}$$

11 $$\begin{aligned} & u_{ij} = \left( {q_{ij} - \sum\limits_{k = 1}^{i - 1} {l_{ik} u_{kj} d_{kk} } } \right)/d_{ii} \\ & \quad i = 1,2,...,n - 1; \, j = i{ + }1,...,n, \\ \end{aligned}$$

where the $l_{ij}$ is the elements of matrix ${\mathbf{L}}$, $d_{ii}$ is the elements of matrix ${\mathbf{D}}$, $u_{ij}$ is the elements of matrix ${\mathbf{U}}$, $q_{ij}$ is the elements of matrix ${\mathbf{Q}}$.

### C-Cholesky decomposition algorithm

Efficient structure is the key to the efficiency of Cholesky decomposition algorithm. From the Cholesky decomposition of Eqs. (9) to (11), we can see that the calculation of each element is complex and the structure is scattered. Therefore, this section will propose an improved C-Cholesky decomposition model by studying the structural relationship between the elements in Cholesky decomposition.

Firstly, the decomposition process of traditional matrix elements is divided into two steps: refresh and column division (cdiv). The cdiv step is to perform the calculation of square root and column division, and the refresh step is the refresh task for multiplication. The cdiv and refresh iteration formula of matrix elements are as follows

12 $$\left\{ \begin{gathered} {\text{cdiv: }}{\mathbf{D}}_{i,i}^{i} = {\mathbf{Q}}_{i,i} , \, {\mathbf{L}}_{r,i}^{i} = {\mathbf{Q}}_{r,i}^{i} /{\mathbf{D}}_{i,i}^{i} \hfill \\ {\text{refresh: }}{\mathbf{Q}}_{r,c}^{i + 1} = {\mathbf{Q}}_{r,c}^{i} - {\mathbf{L}}_{r,i}^{i} {\mathbf{U}}_{i,c}^{i} , \hfill \\ \end{gathered} \right.$$

where the $i$ represents the number of iterations, that is, the number of steps. First, the elements of the first column of matrix ${\mathbf{Q}}$ are reduced by taking its diagonal element ${\mathbf{Q}}_{1,1}$ as the reduction element, and the elements of the first column of matrix ${\mathbf{L}}$ are obtained. Then, the elements in the first column of the ${\mathbf{L}}$ matrix are used to refresh and iterate the elements satisfying $r > c > i$ in the matrix ${\mathbf{Q}}$.

**Figure 1:**
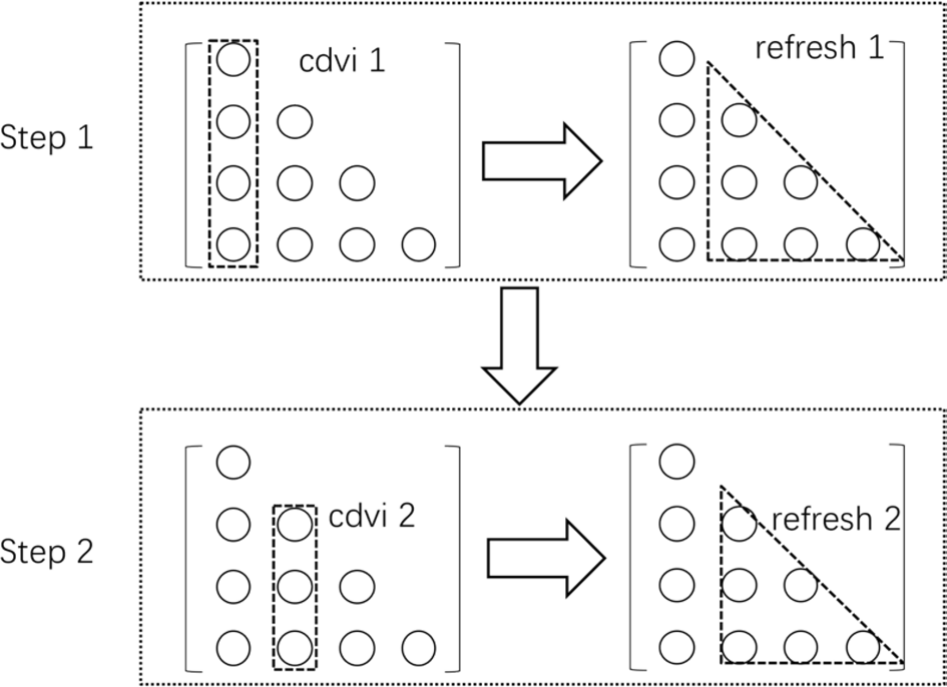
Process of decomposition.

In order to more clearly explain the relationship between column division and refresh, taking four-dimensional matrix as an example, the Cholesky decomposition process based on cdiv and refresh steps is given, as shown in Fig. 1.

**Figure 2:**
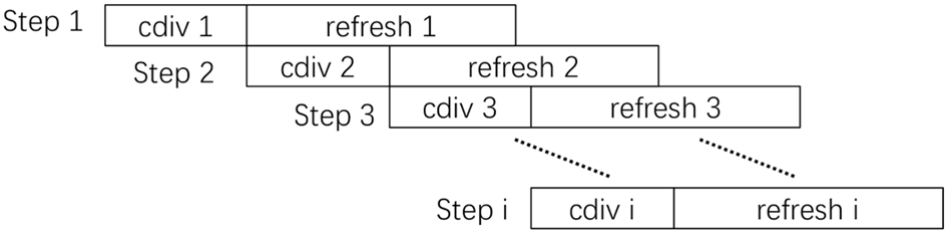
Task scheduling.

When decomposing based on Fig. 1, because matrix ${\mathbf{Q}}$ is symmetrical and equal to the main diagonal elements, only half of the matrix elements need to be calculated and stored. The matrix elements are decomposed column by column. After one column is decomposed, the remaining matrix columns are updated by the elements in the decomposed column. With the decomposition of matrix columns from left to right, the number of elements to be decomposed and updated will continue to decrease, and the processing time of column reduction and update will also decrease accordingly. In order to further improve the efficiency of the decomposition process, the two steps are processed in parallel. The task arrangement of the whole decomposition process is shown in Fig. 2.

As can be seen from Fig. 2, column reduction 2 has started before update 1 is completed, that is, in the second step of column reduction, the reduction step is calculated in parallel with the previous update step, thereby reducing the time required for decomposition. The dimension of the matrix is shown in this figure, and this step is to handle the column element of the matrix with the pointer. In addition, the algorithm realizes the pipelined operation between different columns, while the iterative operation in Eq. (12) is carried out in the same column. Therefore, it has high practicability in the actual programming operation.

## Results and discussion

Usually, in order to test the reliability of the integer ambiguity resolution algorithm, a lot of experiments are needed to verify, and the measured data has a certain particularity because it is affected by the external environment. In addition, the simulation experiment can simulate the floating point solution and corresponding covariance matrix under different conditions, and the dimension is controllable. Therefore, a simulation platform for integer ambiguity resolution is should be built based on the C language platform. After the feasibility of the proposed algorithm is verified by the simulation results, it is further verified by the measured data. The computer platform processor used is Intel (R) core (TM) i7-8750h with 16 GB memory CPU@2.20 Hz. In order to test the reliability of the integer ambiguity resolution algorithm, a lot of experimental verification is needed. If the experiment is carried out only based on the measured data, because the measured data has certain particularity in the interference of external factors such as the strength of satellite signals, weather conditions and environmental conditions, this experiment adopts the combination of simulation experiment and measured experiment. After the simulation experiment results verify the feasibility of the proposed decorrelation algorithm, Further verification is carried out through the measured data.

In this paper, the cumulative distribution function (CDF) graph and the columnar comparison graph of the solution time are used to compare the decorrelation performance of the two algorithms. The cumulative distribution function is the integral of the probability density function, which can completely describe the probability distribution of a variable, and visually compare the solution efficiency of the two algorithms; Bootstrap success rate is usually regarded as the lower boundary of integer least-squares ambiguity resolution success rate, which is an index to evaluate the quality of an integer ambiguity resolution method, in which $P$ is calculated according to the following formula^26,30^.

13 $$P = \mathop \Pi \limits_{i = 1}^{n} \left[ {2\phi \left( {\frac{i}{{2\sqrt {d_{i} } }}} \right) - 1} \right],$$

where the $d_{i}$ is the element in the decomposed matrix ${\mathbf{D}}$, and $\phi \left( x \right)$ is the standard normal distribution function.

14 $$\phi \left( x \right) = \int\limits_{ - \infty }^{\infty } {\frac{1}{{\sqrt {2\pi } }}} \exp \left( { - \frac{1}{2}z^{2} } \right)dz.$$

### Simulation experiment analysis

The simulation experiment is designed with reference to the parameter setting from reference (15). First, the floating-point solution $\hat{a}$ is generated randomly:

15 $${\hat{\mathbf{a}}} = 100 \times {\text{randn}}(n,1),$$

where the ${\text{randn}}(n,1)$ is a random function that generates $n$ random numbers that conform to normal distribution, $n$ is the dimension of covariance matrix ${\mathbf{Q}}_{{\hat{a}}}$.

The covariance matrix ${\mathbf{Q}}_{{\hat{a}}}$ is constructed based on the following four cases.

1. ${\mathbf{Q}}_{{\hat{a}}} = {\mathbf{L}}^{{\text{T}}} {\mathbf{DL}}$ and ${\mathbf{D}} = {\text{diag}}(d_{i} )$. Where the ${\mathbf{L}}$ is the unit lower triangular matrix. The element $l_{ij} \, \left( {i > j} \right)$ in each matrix ${\mathbf{L}}$ is a random number generated by the function ${\text{randn}}(n,1)$. $d_{i}$ is a random number uniformly distributed in the interval (0,1) returned by the rand function.
2. ${\mathbf{D}} = {\text{diag}}(200,200,200,0.1,0.1, \ldots ,0.1)$. The construction of covariance matrix is the same as that of case 1, which takes into account the large magnitude difference between the first three standard deviations of GNSS covariance matrix ${\mathbf{Q}}_{{\hat{a}}}$ and the subsequent standard deviations.
3. ${\mathbf{UDU}}^{{\text{T}}}$ decomposition of ${\mathbf{Q}}_{{\hat{a}}}$, ${\mathbf{U}}$ is an orthogonal matrix obtained by QR decomposition of the random matrix generated by ${\text{randn}}(n,n)$, ${\mathbf{D}} = {\text{diag}}(d_{i} )$, $d_{i} = {\text{rand}}$.
4. ${\mathbf{UDU}}^{{\text{T}}}$ decomposition of ${\mathbf{Q}}_{{\hat{a}}}$, the construction of ${\mathbf{U}}$ is the same as that of case 3, in the matrix ${\mathbf{D}}$, $d_{1} = 2^{{{ - }\tfrac{n}{4}}}$, $d_{n} = 2^{{\tfrac{n}{4}}}$, the other diagonal elements are randomly distributed between $d_{1}$ and $d_{n}$, and $n$ is the dimension of the ${\mathbf{Q}}_{{\hat{a}}}$ matrix.

For the above four cases, the Cholesky algorithm and the C-Cholesky algorithm proposed in this paper are used for simulation experiments. In order to illustrate the universality of the algorithm, the simulation dimension is from five dimensions to forty dimensions. To avoid accidental situations, each simulation experiment is conducted for 100 times to take the average value.

**Figure 3:**
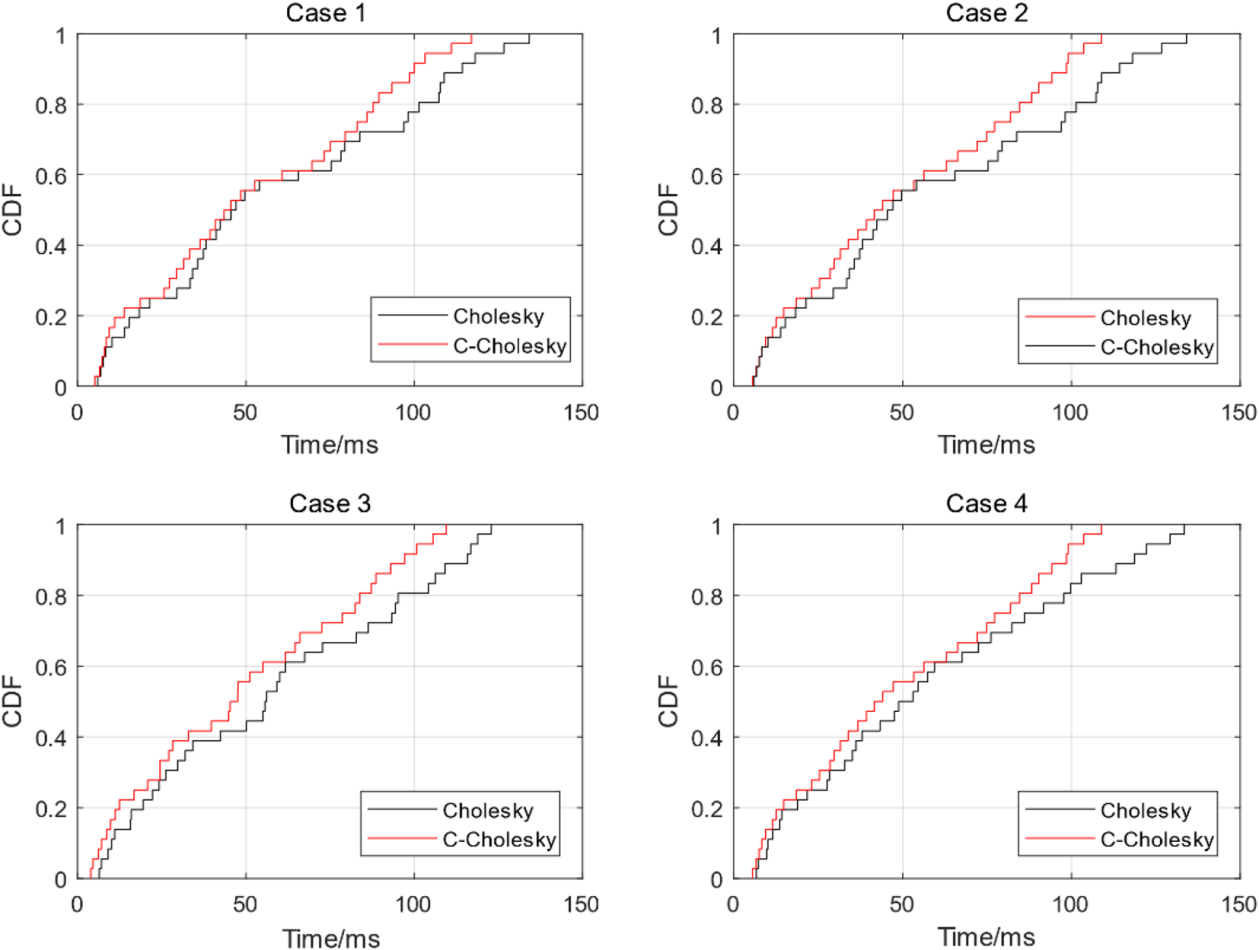
Four case probability distribution function comparison.

From the probability distribution comparison diagram of the four cases in Fig. 3, the probability distribution of the solution time of the two algorithms in 5–40 dimensions can be obtained. It can be seen that the resolution time distribution range of the C-Cholesky algorithm proposed in this paper is smaller than that of the traditional Cholesky algorithm. In order to further clearly compare the solution time of the two algorithms, the solution time comparison histogram and the average solution time comparison in four cases are given.

**Figure 4:**
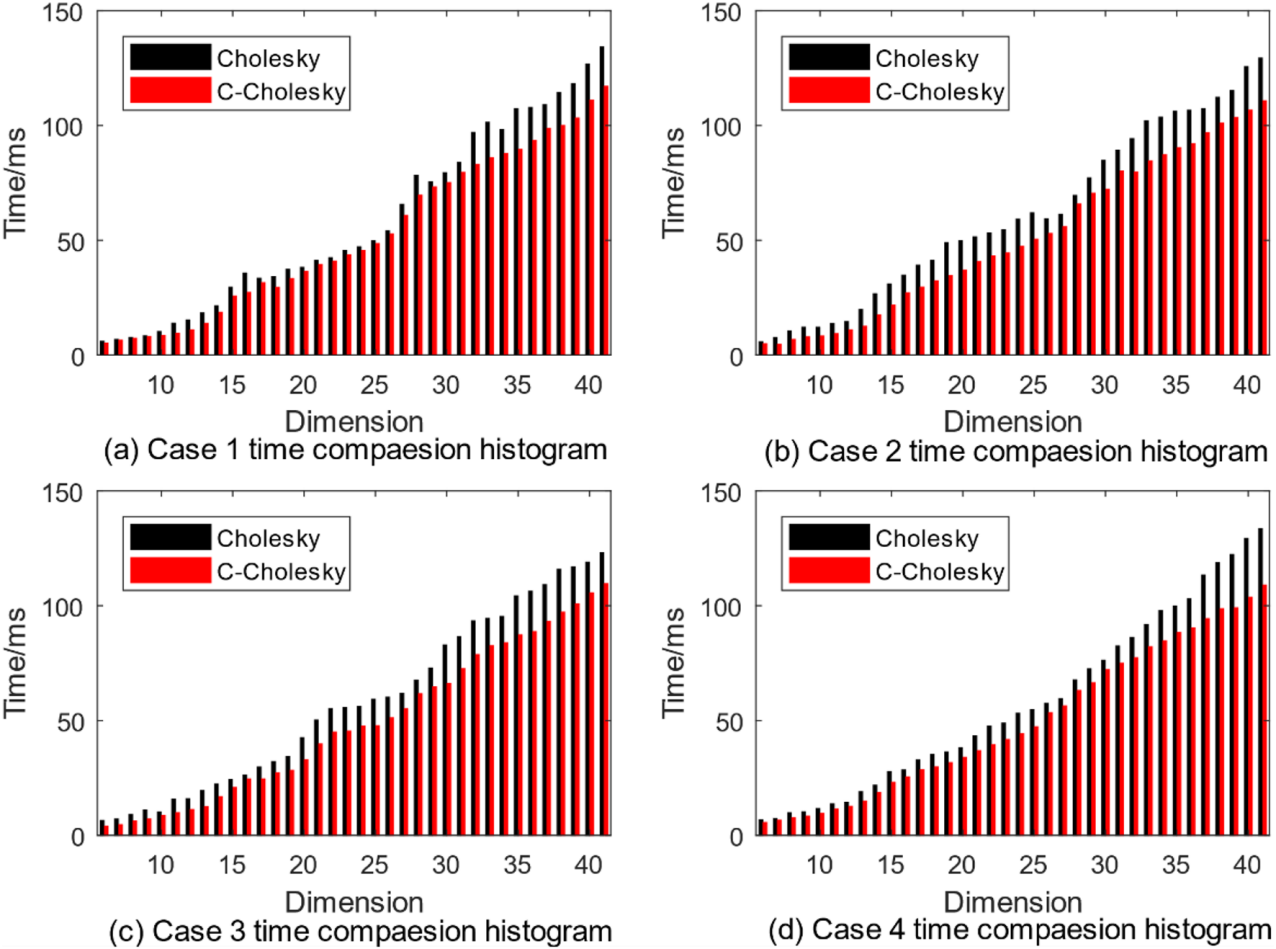
Simulation time comparison histogram.

It can be seen from the simulation experiment comparison histogram in Fig. 4 that the solution time of the two algorithms increases as the dimension increases. In four cases, the solution time of C-Cholesky algorithm is less than that of traditional Cholesky algorithm as a whole, and the solution efficiency of C-Cholesky algorithm is gradually improved during the growth of dimension from 5 to 40 dimensions. By comparing the average solution time of the four cases from Table 1, we can get the solution time of the simulation experiment in each case. Compared with the traditional Cholesky algorithm, C-Cholesky algorithm improves the solution efficiency by about 10%—16%.

**Table 1:** Comparison of average solution time.

Simulation case	Cholesky (ms)	C-Cholesky (ms)	Improve efficiency (%)
Case 1	58.1040	51.9450	10.59
Case 2	60.8511	51.1212	15.99
Case 3	58.0507	48.9264	15.71
Case 4	57.5028	49.6761	13.61

### Analysis of measured data

**Figure 5:**
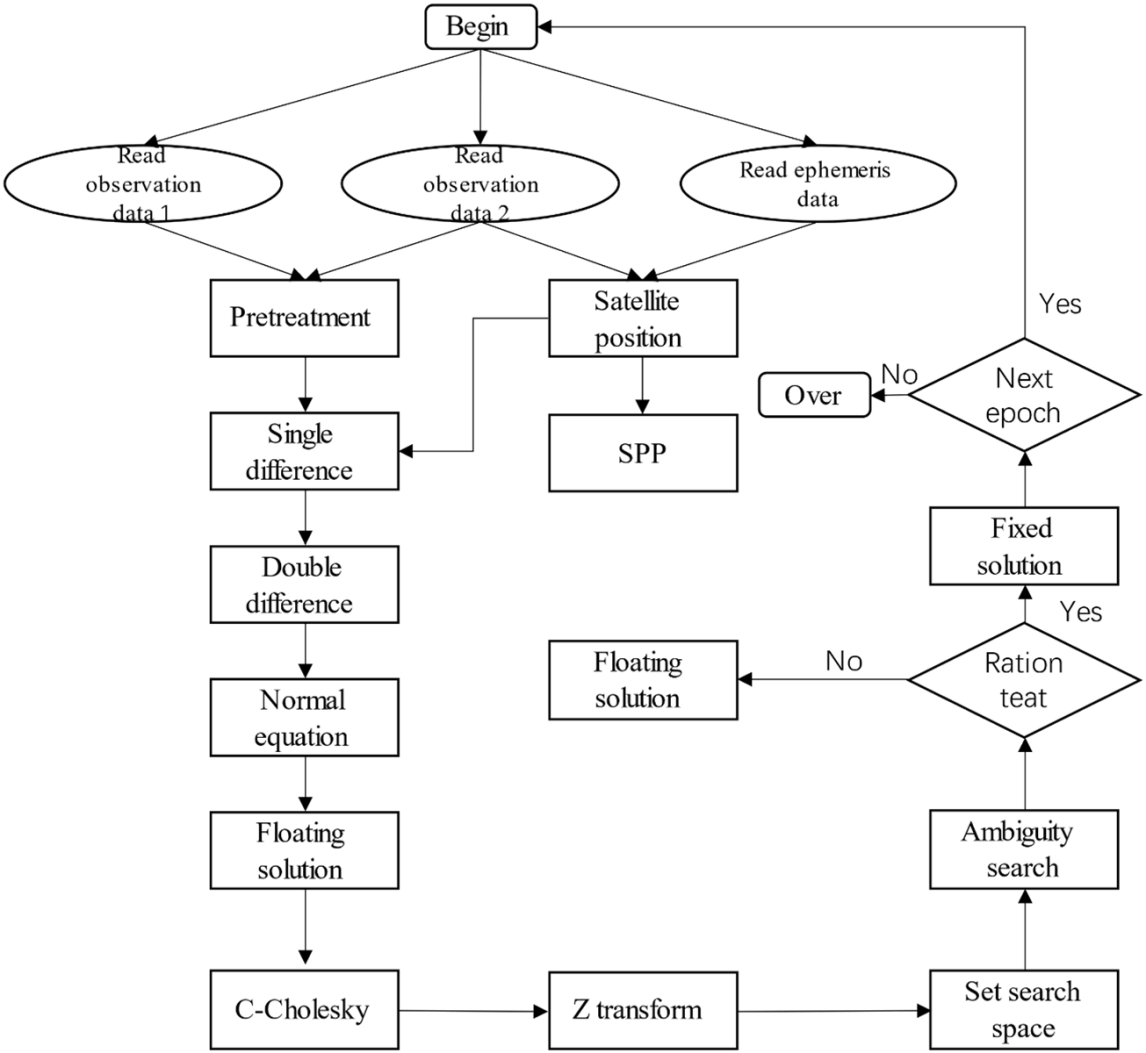
Flow chart of baseline solution.

The baseline solution flow is shown in Fig. 5.

**Figure 6:**
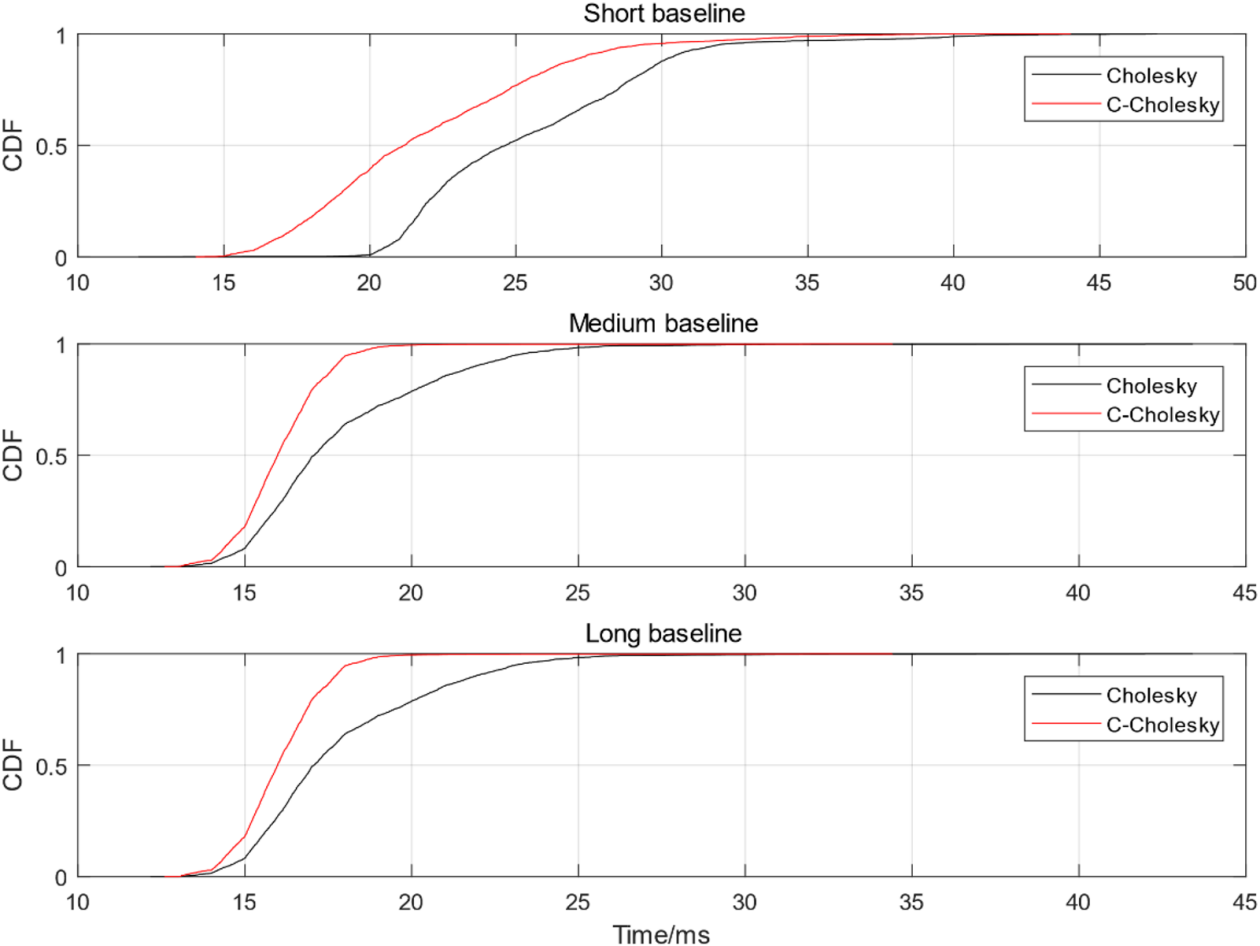
Short baseline probability distribution function comparison.

**Figure 7:**
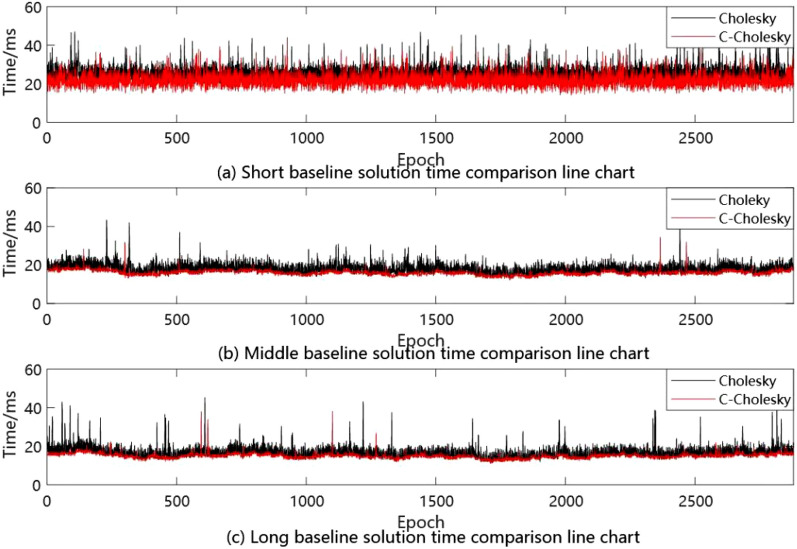
Measured experimental time comparison line chart.

The measured experiments were divided into three groups: short baseline, medium baseline and long baseline. The short baseline group adopts the static observation data collected by School of Surveying and Land Information Engineering, Henan Polytechnic University. The baseline is 50 m long and the sampling interval is 1 s. 3000 epochs are intercepted for experimental analysis of the solution time. The middle baseline group adopts the IGS data of two stations in Hong Kong, HKSL and HKWS. The baseline length of the two stations is 42.51 km, the sampling interval is 30 s, and a total of 2880 epochs are used for data analysis; The long baseline group adopts the IGS data of two stations, HKSL in Hong Kong and JFNG in Wuhan. The baseline length of the two stations is 903.26 km, the sampling interval is 30 s, and a total of 2880 epochs are used for data analysis. The baseline solution success rate of short, medium and long baseline solutions using Cholesky and C-Cholesky algorithms is shown in Table 2, the comparison of probability distribution functions are shown from Fig. 6, and the histogram of measured experimental time comparison is shown in Fig. 7.

**Table 2:** Baseline solution success rate of C-Cholesky and Cholesky adjustment with different baseline length.

Case	Method	Success rate
Short baseline	C-Cholesky	0.9998
	Cholesky	0.9998
Medium baseline	C-Cholesky	0.9896
	Cholesky	0.9896
Long baseline	C-Cholesky	0.9896
	Cholesky	0.9896

It can be seen from Table 2 that the baseline solution success rate of the improved C-Cholesky algorithm for solving integer ambiguity remains unchanged, that is, the algorithm does not affect the accuracy of the solution. It can be seen from Fig. 6 that in the process of solving the measured data of short baseline, the solving time of the improved C-Cholesky algorithm is significantly lower than that of the traditional Cholesky algorithm, and the fastest single epoch solving efficiency can be achieved by 15 ms, and the overall solving efficiency is maintained at about 15–37 ms; The solution efficiency of traditional Cholesky algorithm is about 19–43 ms. As can be seen from the medium baseline probability distribution function diagram in Fig. 6, the overall solution time of C-Cholesky algorithm is about 13–20 ms, while the solution time of Cholesky algorithm is about 13–30 ms. The long baseline probability distribution diagram in Fig. 6 shows that the overall solution time of C-Cholesky algorithm is about 12–20 ms, while the solution time of Cholesky algorithm is about 12–35 ms. In order to further prove the improvement of its solution efficiency, the measured experiment comparison line chart under three baseline lengths are given, as shown in Fig. 7.

From the time comparison line chart of the measured experiment in Fig. 7, it can be seen that under the three baseline lengths, the solution time of C-Cholesky algorithm is better than that of Cholesky algorithm, and it is more stable. After calculation, the C-Cholesky algorithm improves the solution efficiency by 14%, 10% and 12% respectively under the three baseline lengths. To sum up, the C-Cholesky algorithm proposed in this paper is superior to the traditional Cholesky algorithm in solving the ambiguity of the whole cycle on the premise of ensuring the same accuracy.

## Conclusions

In this study, the traditional Cholesky algorithm based on the two-step parallel processing principle of column reduction and update is appropriately improved, and a more superior C-Cholesky algorithm is obtained. Using the simulation experimental data of these four different construction cases about covariance matrix and the measured data of short, medium and long baselines, the solution time of covariance matrix reduction correlation using C-Cholesky algorithm is analyzed. The results confirm the superiority and effectiveness of this method. This method can improve the solution efficiency on the premise of ensuring the accuracy of integer ambiguity resolution.

## Supplementary Information

Supplementary Information.

## Data Availability

The authors confirm that the data supporting the findings of this study are available within the article and its supplementary materials. The supplementary materials including simulation data and raw data of the experiments. And the details on how to access it in the document description file. The short baseline datasets analyzed in this study are managed by the School of Surveying and Land Information Engineering, Henan Polytechnic University and can also be available on request from the corresponding author. The medium baseline and long baseline datasets analyzed in this study can also be download from the IGS Data Center of Wuhan University, website link: http://www.igs.gnsswhu.cn/. All the raw data used are standard RINEX format data, including ephemeris data and observation data.
