# Supplementary material for: Improved GNSS integer ambiguity resolution method based on the column oriented Cholesky decomposition
Source: Sci Rep. 2023 Mar 17;13:4454. doi: 10.1038/s41598-023-31635-3 (PMC10023790; doi:10.1038/s41598-023-31635-3)
Supplement: Supplementary file 1 — Supplementary Information. [file 41598_2023_31635_MOESM1_ESM.zip › supplement materials of the manuscript/Document description.doc]

Data Description

The supplementary materials including simulation data and raw data of the experiments. And the details on how to access them in the corresponding document description files.

The file descriptions corresponding to each picture in this article are in the corresponding folder (Figure 3, Figure 4, Figure 5, and Figure 6). For specific data format descriptions and other instructions, see the corresponding Doc file.
