# Supplementary material for: Improved GNSS integer ambiguity resolution method based on the column oriented Cholesky decomposition
Source: Sci Rep. 2023 Mar 17;13:4454. doi: 10.1038/s41598-023-31635-3 (PMC10023790; doi:10.1038/s41598-023-31635-3)
Supplement: Supplementary file 1 — Supplementary Information. [file 41598_2023_31635_MOESM1_ESM.zip › supplement materials of the manuscript/Figure 3/Figure 3 Data Description.doc]

- Simulation experiment/Excel Tables Figure 3-sumulation 1-4 are simulation data，

the corresponding results of this data are shown in Figure 3。

- The format in the xlsx file is described as follows :Columns 1 to 36 in the simulation 1 table are the floating point solutions generated according to the simulation data construction formula (15) in the paper.

Columns 37 to 76 are Lt matrices generated according to the simulation data structure (1) in the paper.

Columns 77 to 116 are D-matrices generated according to the simulation data structure (1) in the paper.

Columns 157 to 966 are the covariance matrix generated according to the simulation data structure (1) in the paper.

Columns 1 to 36 in the simulation 2 table are the floating point solutions generated according to the simulation data construction formula (15) in the paper.

Columns 37 to 76 are Lt matrices generated according to the simulation data structure (2) in the paper.

Columns 77 to 116 are D-matrices generated according to the simulation data structure (2) in the paper.

Columns 157 to 966 are the covariance matrix generated according to the simulation data structure (2) in the paper.

Columns 1 to 36 in the simulation 3 table are the floating point solutions generated according to the simulation data construction formula (15) in the paper.

Columns 37 to 76 are D-matrices generated according to the simulation data structure (3) in the paper.

Columns 77 to 925 are U-matrices generated according to the simulation data structure (3) in the paper.

Columns 926 to 1735 are the covariance generated according to the simulation data in the paper (3).

Columns 1 to 36 in the simulation 4 table are the floating point solutions generated according to the simulation data construction formula (15) in the paper.

Columns 37 to 846 are D-matrices generated according to the simulation data structure (4) in the paper.

Columns 847 to 1695 are U-matrices generated according to the simulation data structure (4) in the paper.

Columns 1697 to 2505 are the covariance generated according to the simulation data (4) in the paper.

- The simulation solution time is shown in Simulation solution time.xlsx。
