# Supplementary material for: Improved GNSS integer ambiguity resolution method based on the column oriented Cholesky decomposition
Source: Sci Rep. 2023 Mar 17;13:4454. doi: 10.1038/s41598-023-31635-3 (PMC10023790; doi:10.1038/s41598-023-31635-3)
Supplement: Supplementary file 1 — Supplementary Information. [file 41598_2023_31635_MOESM1_ESM.zip › supplement materials of the manuscript/Figure 5/rinex302.pdf]

RINEX

The Receiver Independent Exchange Format

Version 3.02

International GNSS Service (IGS), RINEX Working Group and Radio Technical Commission for Maritime Services Special Committee 104 (RTCM-SC104),

April 3, 2013

Acknowledgment: RINEX Version 3.02 is based on RINEX Version 3.01 which was developed by: Werner Gurtner, Astronomical Institute of the University of Bern, Switzerland and Lou Estey, UNAVCO, Boulder Colorado, USA.

Table of Contents

0. REVISION HISTORY 1
1. THE PHILOSOPHY AND HISTORY OF RINEX 4
2. GENERAL FORMAT DESCRIPTION 6
3. BASIC DEFINITIONS 7
3.1 Time 7
3.2 Pseudo-Range: 7
3.3 Phase 7
3.4 Doppler 8
3.5 Satellite numbers 8
Figure 1: Satellite numbers and Constellation Identifiers 8
4. THE EXCHANGE OF RINEX FILES: 9
Figure 2: Recommended filename parameters. 9
Table 1: Description of filename parameters 10
5. RINEX VERSION 3 FEATURES 11
5.1 Observation codes 11
Table 2 : RINEX Version 3.02 GPS observation codes 12
Table 3 : RINEX Version 3.02 GLONASS observation codes 13
Table 4 : RINEX Version 3.02 Galileo observation codes 13
Table 5 : RINEX Version 3.02 SBAS observation codes 14
Table 6 : RINEX Version 3.02 QZSS observation codes 14
Table 7 : RINEX Version 3.02 BDS observation codes 15
5.2 Satellite system-dependent list of observables 15
5.3 Marker type 16
Table 8: Proposed marker type keywords 16
5.4 Half-wavelength observations, half-cycle ambiguities 17
5.5 Scale factor 17
5.6 Information about receivers on a vehicle 17
5.7 Signal strengths 18
Table 9: Standardized S/N indicators 18
5.8 Date/time format in the PGM / RUN BY / DATE header record 18

5.9 Antenna phase center header record 19
5.10 Antenna orientation 19
5.11 Observation data records 19
Table 10: Example for a list of observation types 19
Table 11: Example for observation data records 19
5.12 Ionosphere delay as pseudo-observables 20
5.13 Channel numbers as pseudo-observables 20
5.14 Corrections of differential code biases (DCBs) 21
5.15 Corrections of antenna phase center variations (PCVs) 21
5.16 Navigation message files 22
6. ADDITIONAL HINTS AND TIPS 23
6.1 Versions 23
6.2 Leading blanks in CHARACTER fields 23
6.3 Variable-length records 23
6.4 Blank fields 23
6.5 Order of the header records, order of data records 23
6.6 Missing items, duration of the validity of values 24
6.7 Unknown / Undefined observation types and header records 24
6.8 Event flag records 24
6.9 Receiver clock offset 25
6.10 Two-digit years 25
6.11 Fit interval (GPS navigation message file) 25
6.12 Satellite health (GPS navigation message file) 26
6.13 Transmission time of message (GPS navigation message file) 26
6.14 Antenna references, phase centers 26
7. RINEX UNDER ANTISPOOFING (AS) 27
8. DEALING WITH DIFFERENT SATELLITE SYSTEMS 28
8.1 Time system identifier 28
Table 12: Relations between GPS, QZSS, GST, GAL and BDS weeks 29
8.2 Pseudorange definition 31
8.3 RINEX navigation message files 31
8.3.1 RINEX navigation message files for GLONASS 32

8.3.2 RINEX navigation message files for Galileo 32
8.3.3 RINEX navigation message files for GEO satellites 33
8.3.4 RINEX navigation message files for QZSS L1-SAIF 34
8.3.5 RINEX navigation message files for BDS 34
8.4 RINEX observation files for GEO satellites 35
9. MODIFICATIONS FOR VERSION 3.01 and 3.02 36
9.1 Phase Cycle Shifts 36
9.2 Galileo: BOC-Tracking of an MBOC-Modulated Signal 37
9.3 BDS Satellite System Code 38
9.4 New Observation Codes for GPS L1C and BDS 38
9.5 Header Records for GLONASS Slot and Frequency Numbers 38
9.6 GNSS Navigation Message File: Leap Seconds Record 38
9.7 Clarifications in the Galileo Navigation Message File: 38
9.8 RINEX Meteorological Data Files 38
9.9 Added Quasi-Zenith Satellite System (QZSS) Version 3.02 38
9.10 Added GLONASS Mandatory Code-Phase Alignment Header Record 39
9.11 Added BDS system (Replaces Compass) 39
10. REFERENCES 40
APPENDIX: RINEX FORMAT DEFINITIONS AND EXAMPLES 1
A 1 RINEX File name description 1
A 2 GNSS Observation Data File -Header Section Description 5
A 3 GNSS Observation Data File -Data Record Description 12
A 4 GNSS Observation Data File - Example 14
A 5 GNSS Navigation Message File – Header Section Description 16
A 6 GNSS Navigation Message File – GPS Data Record Description 18
A 7 GPS Navigation Message File – Example 19
A 8 GNSS Navigation Message File – GALILEO Data Record Description 20
A 9 GALILEO Navigation Message File – Example 22
A 10 GNSS Navigation Message File – GLONASS Data Record Description 23
A 11 GNSS Navigation Message File – Example: Mixed GPS / GLONASS 24
A 12 GNSS Navigation Message File – QZSS Data Record Description 25
A 13 GNSS Navigation Message File – BDS Data Record Description 27

A 14 GNSS Navigation Message File – SBAS Data Record Description 29
A 15 SBAS Navigation Message File -Example 30
A 16 Meteorological Data File -Header Section Description 31
A 17 Meteorological Data File -Data Record Description........ 33
A 18 Meteorological Data File – Example 33
A 19 Reference Code and Phase Alignment by Constellation and Frequency Band........ 34

0. REVISION HISTORY

	**Version 3.00**
02 Feb 2006	A few typos and obsolete paragraphs removed.
08 Mar 2006	Epochs of met data of met files version 2.11 are in GPS time only (Table A15).
31 Mar 2006	DCB header record label corrected in Table A6**:** **`SYS / DCBS APPLIED.`**
June 2006	Filenames for mixed GNSS nav mess files.
10 Aug 2006	Table A3: Error in format of ***`EPOCH`*** record:<br>One 6X removed. Trailing 3X removed.
12 Sep 2006	GNSS navigation message files version 3.00 included (including Galileo).<br>Table A4: Example of the kinematic event was wrong (kinematic event record).<br>**`SYS / DCBS APPLIED`** header record simplified.<br>Tables A6 and A8: Clarification for adjustment of "Transmission time of message".
03 Oct 2006	Table A11: Mixed GPS/GLONASS navigation message file
26 Oct 2006	Table A4: Removed obsolete antispoofing flag<br>Tables A6/8/10: Format error in ***`SV / EPOCH / SV CLK`***:<br>Space between svn and year was missing<br>Half-cycle ambiguity flag (re-)introduced (5.4 and Table A4).<br>Clarification of reported GLONASS time (8.1).<br>New header record **`SYS / PCVS APPLIED`**<br>New Table 10: Relations between **`GPS`**, GST, and **`GAL`** weeks<br>Recommendation to avoid storing redundant navigation messages (8.3)
14 Nov 2006	Tables A6/10/12: Format error in<br>***`BROADCAST ORBIT - n`***`: 3X` → `4X.` Examples were OK.
21 Nov 2006	Marker type **`NON_PHYSICAL`** added
19-Dec-2006	Table A4: Example of **`SYS / DCBS APPLIED`** was wrong.
13-Mar-2007	Paragraph 3.3: Leftover from RINEX version 2 regarding wavelength factor for squaring- type receiver removed and clarified.
14-Jun-2007	Paragraph 5.11: Clarification regarding the observation record length
28-Nov-2007	Frequency numbers for GLONASS –7..+12 (***`BROADCAST ORBIT -`***
	**Version 3.01**
22-Jun-2009	Phase cycle shifts<br><br>Galileo: BOC-tracking of an MBOC-modulated signal<br>Compass satellite system: Identifier and observation codes<br>Code for GPS L1C<br>Header records for GLONASS slot and frequency numbers<br>Order of data records

	Galileo nav mess record ***BROADCAST ORBIT - 5***:<br>Bits ¾ reserved for Galileo internal use
	**Version 3.02 – IGS and RTCM-SC104**
19-Nov-2011	Added Quasi Zenith Satellite System (QZSS) Constellation<br>Updated text, tables and graphics<br>Added Appendix Table 19 phase alignment table
21-Jan-2012	Split the Constellation table into a table for each GNSS<br>Added QZSS to the documentation<br>Edited text to improve clarity<br>Corrected sign in the phase alignment table, removed QZSS P signals<br>Removed
9-May-2012	Edited text to improve clarity,<br>Updated phase alignment table,<br>Changed Met `PGM / RUN BY / DATE` to support 4digit year as in all other records also changed format to support 4 digit year for met. Observation record,<br>Changed SYS / PHASE SHIFTS to SHIFT
29-Nov-2012	Changed Table1 and 2 to Figure 1 and 2. Updated all Table numbers<br>Changed file naming convention, Section 4. Added Appendix Table A1 and increased all updated all Appendix numbers<br>Removed the option of supporting unknown tracking mode from Section 5.1.<br>Harmonized L1C(new) signal identifiers for QZSS and GPS See : Table 2 and 6.<br>Updated BeiDou System (BDS) (was Compass) information through ou document added new BDS ephemeris definition to Appendix. (Based on input from the BDS Office)<br>Corrected GLONASS SLOT/FRQ format in section 9.5, changed message status from optional to mandatory (See: Appendix Table A2).<br>Added new mandatory GLONASS Code Phase Bias header record See section 9.10
11-Mar-2013	Updated Sections: 4.x, made .rnx the file name extension and updated Figure 2; 9.1 to clarify the use of the phase alignment header; A1 Edited to reflect file extension of *.rnx; A13 - BDS ephemeris changed AODC to IODC and AODE to IODE (as indicated by BDS Authority and new ICD); Appendix Table 19 (Changed GLONASS Reference Signals to C1-C2) and explicitly identified reference signal for all constellations and frequencies.
26-Mar-2013	Changed BeiDou to BDS for conform to ICD.<br>In table 7 changed BDS signals from : C2x, C1x to more closely reflect existing bands in tables 2-6 and Appendix Tables A2 and A19.<br>Updated Section 8.1: First paragraph updated to indicate current number of leap seconds; added a row to Table 12 to show the relationship between GPS week and BDT week. Added a table to show the approximate relationship of BDT to GPS time.

Changed order of file type: from OG to GO etc in Appendix Table 1.
Updated Appendix table A19 to show X signals and indicate that the X phase is to be aligned to the frequencies reference signal.
Fixed a few small typos in A19 for GPS: L1C-D/P and D+P.

1. THE PHILOSOPHY AND HISTORY OF RINEX

The first proposal for the ***Receiver Independent Exchange Format RINEX*** was developed by the Astronomical Institute of the University of Berne for the easy exchange of the Global Positioning System (GPS) data to be collected during the first large European GPS campaign EUREF 89, which involved more than 60 GPS receivers of 4 different manufacturers. The governing aspect during the development was the following fact:

Most geodetic processing software for GPS data use a well-defined set of observables:

- the carrier-phase measurement at one or both carriers (actually being a measurement on the beat frequency between the received carrier of the satellite signal and a receiver-generated reference frequency).
- the pseudorange (code) measurement, equivalent to the difference of the time of reception (expressed in the time frame of the receiver) and the time of transmission (expressed in the time frame of the satellite) of a distinct satellite signal.
- the observation time being the reading of the receiver clock at the instant of validity of the carrier-phase and/or the code measurements.

Usually the software assumes that the observation time is valid for both the phase **and** the code measurements, **and** for all satellites observed.

Consequently all these programs do not need most of the information that is usually stored by the receivers: They need phase, code, and time in the above mentioned definitions, and some station-related information like station name, antenna height, etc.

Up till now two major format versions have been developed and published:

- The original RINEX Version 1 presented at and accepted by the 5th International Geodetic Symposium on Satellite Positioning in Las Cruces, 1989. [Gurtner et al. 1989], [Evans 1989]
- RINEX Version 2 presented at and accepted by the Second International Symposium of Precise Positioning with the Global Positioning system in Ottawa, 1990, mainly adding the possibility to include tracking data from different satellite systems (GLONASS, SBAS). [Gurtner and Mader 1990a, 1990b], [Gurtner 1994].

Several subversions of RINEX Version 2 have been defined:

- Version 2.10: Among other minor changes allowing for sampling rates other than integer seconds and including raw signal strengths as new observables. [Gurtner 2002]

- Version 2.11: Includes the definition of a two-character observation code for L2C pseudoranges and some modifications in the GEO NAV MESS files [Gurtner and Estey 2005]

- Version 2.20: Unofficial version used for the exchange of tracking data from spaceborne receivers within the IGS LEO pilot project [Gurtner and Estey 2002]

As spin-offs of this idea of a receiver-independent GPS exchange format other RINEX-like exchange file formats have been defined, mainly used by the International GNSS Service IGS:

- Exchange format for **satellite and receiver clock offsets** determined by processing data of a GNSS tracking network [Ray and Gurtner 1999]
- Exchange format for the complete **broadcast data of space-based augmentation systems** SBAS. [Suard et al. 2004]
- IONEX: Exchange format for **ionosphere models** determined by processing data of a GNSS tracking network [Schaer et al. 1998]
- ANTEX: Exchange format for **phase center variations** of geodetic GNSS antennae [Rothacher and Schmid 2005]

The upcoming European Navigation Satellite System Galileo and the enhanced GPS with new frequencies and observation types, especially the possibility to track frequencies on different channels, ask for a more flexible and more detailed definition of the observation codes.

To improve the handling of the data files in case of “mixed” files, i.e. files containing tracking data of more than one satellite system, each one with different observation types, the record structure of the data record has been modified significantly and, following several requests, the limitation to 80 characters length has been removed.

As the changes are quite significant, they lead to a new RINEX Version 3. The new version also includes the unofficial Version 2.20 definitions for space-borne receivers.

The major change asking for a version 3.01 was the requirement to generate consistent phase observations across different tracking modes or channels, i.e. to apply ¼-cycle shifts prior to RINEX file generation, if necessary, to facilitate the processing of such data.

2. GENERAL FORMAT DESCRIPTION

The RINEX version 3.00 format consists of three ASCII file types:

1. Observation data File
2. Navigation message File
3. Meteorological data File

Each file type consists of a header section and a data section. The header section contains global information for the entire file and is placed at the beginning of the file. The header section contains **header labels in columns 61-80** for each line contained in the header section. These labels are mandatory and must appear exactly as given in these descriptions and examples.

The format has been optimized for minimum space requirements independent from the number of different observation types of a specific receiver or satellite system by indicating in the header the types of observations to be stored for this receiver and the satellite systems having been observed. In computer systems allowing variable record lengths the observation records may be kept as short as possible. Trailing blanks can be removed from the records. There is no maximum record length limitation for the observation records.

Each Observation file and each Meteorological Data file basically contain the data from one site and one session. Starting with Version 2 RINEX also allows including observation data from more than one site subsequently occupied by a roving receiver in rapid static or kinematic applications. Although Version 2 and higher allow to insert header records into the data section it is not recommended to concatenate data of more than one receiver (or antenna) into the same file, even if the data do not overlap in time.

If data from more than one receiver have to be exchanged, it would not be economical to include the identical satellite navigation messages collected by the different receivers several times. Therefore the navigation message file from one receiver may be exchanged or a composite navigation message file created containing non-redundant information from several receivers in order to make the most complete file.

The format of the data records of the RINEX Version 1 navigation message file was identical to the former NGS exchange format. RINEX version 3 navigation message files may contain navigation messages of more than one satellite system (GPS, GLONASS, Galileo, Quasi Zenith Satellite System (QZSS), BeiDou System (BDS) and SBAS).

The actual format descriptions as well as examples are given in the Appendix Tables at the end of the document.

3. BASIC DEFINITIONS

GPS observables include three fundamental quantities that need to be defined: Time, Phase, and Range.

3.1 Time

The time of the measurement is the receiver time of the received signals. It is identical for the phase and range measurements and is identical for all satellites observed at that epoch. For single-system data files it is by default expressed in the time system of the respective satellite system. Otherwise the actual time can (for mixed files must) be indicated in the Start Time header record.

3.2 Pseudo-Range:

The pseudo-range (PR) is the distance from the receiver antenna to the satellite antenna including receiver and satellite clock offsets (and other biases, such as atmospheric delays):

PR = distance + c * (receiver clock offset –satellite clock offset + other biases)

so that the pseudo-range reflects the actual behaviour of the receiver and satellite clocks. The pseudo-range is stored in units of meters.

See also clarifications for pseudoranges in mixed GPS/GLONASS/Galileo/QZSS/BDS files in chapter 8.1.

3.3 Phase

The phase is the carrier-phase measured in whole cycles. The half-cycles measured by squaring-type receivers must be converted to whole cycles and flagged by the respective observation code (see Table 2 and paragraph 5.4, GPS only).

The phase changes in the same sense as the range (negative doppler). The phase observations between epochs must be connected by including the integer number of cycles.

The observables are not corrected for external effects like atmospheric refraction, satellite clock offsets, etc.

If necessary phase observations are corrected for phase shifts needed to guarantee consistency between phases of the same frequency and satellite system based on different signal channels (See Section 9.1 and Appendix A19).

If the receiver or the converter software adjusts the measurements using the real-time-derived receiver clock offsets dT(r), the consistency of the 3 quantities phase / pseudo-range / epoch must be maintained, i.e. the receiver clock correction should be applied to all 3 observables:

Time (corr)	=	Time(r)	-	dT(r)
PR (corr)	=	PR (r)	-	dT(r)*c
phase (corr)	=	phase (r)	-	dT(r)*freq

3.4 Doppler

The sign of the doppler shift as additional observable is defined as usual: Positive for approaching satellites.

3.5 Satellite numbers

Starting with RINEX Version 2 the former two-digit satellite numbers **nn** are preceded by a one-character system identifier **s**:, see Figure 1.

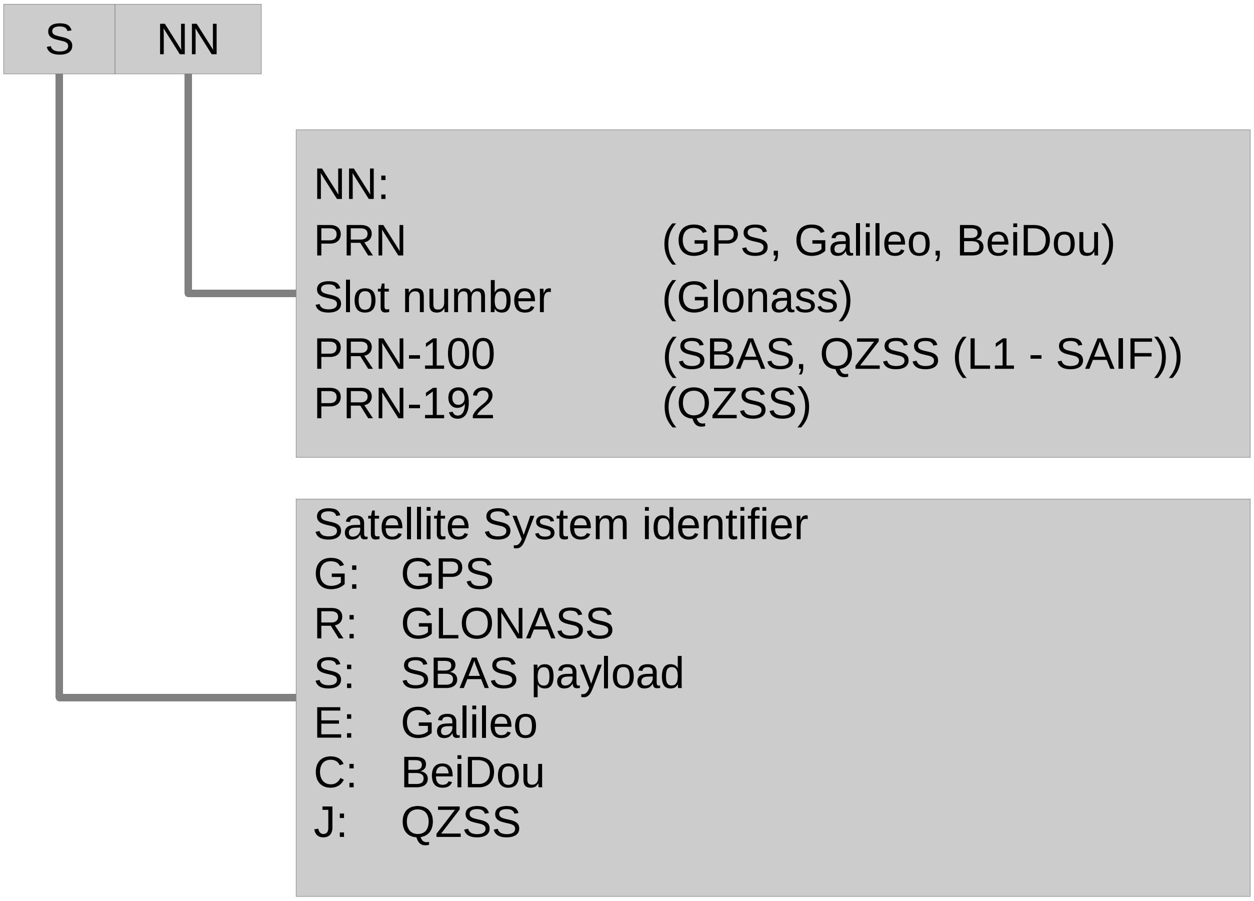

Figure 1: Satellite numbers and Constellation Identifiers

The same satellite system identifiers are also used in all header records when appropriate.

4. THE EXCHANGE OF RINEX FILES:

The original RINEX file naming convention was implemented in the MS-DOS era when file names were restricted to 8.3 characters. Modern operating systems typically support 255 character file names. The goal of the new file naming convention is to be more: descriptive, flexible and extensible than the RINEX 2.11 file naming convention. Figure 2 below lists the elements of the RINEX 3.02 file naming convention.

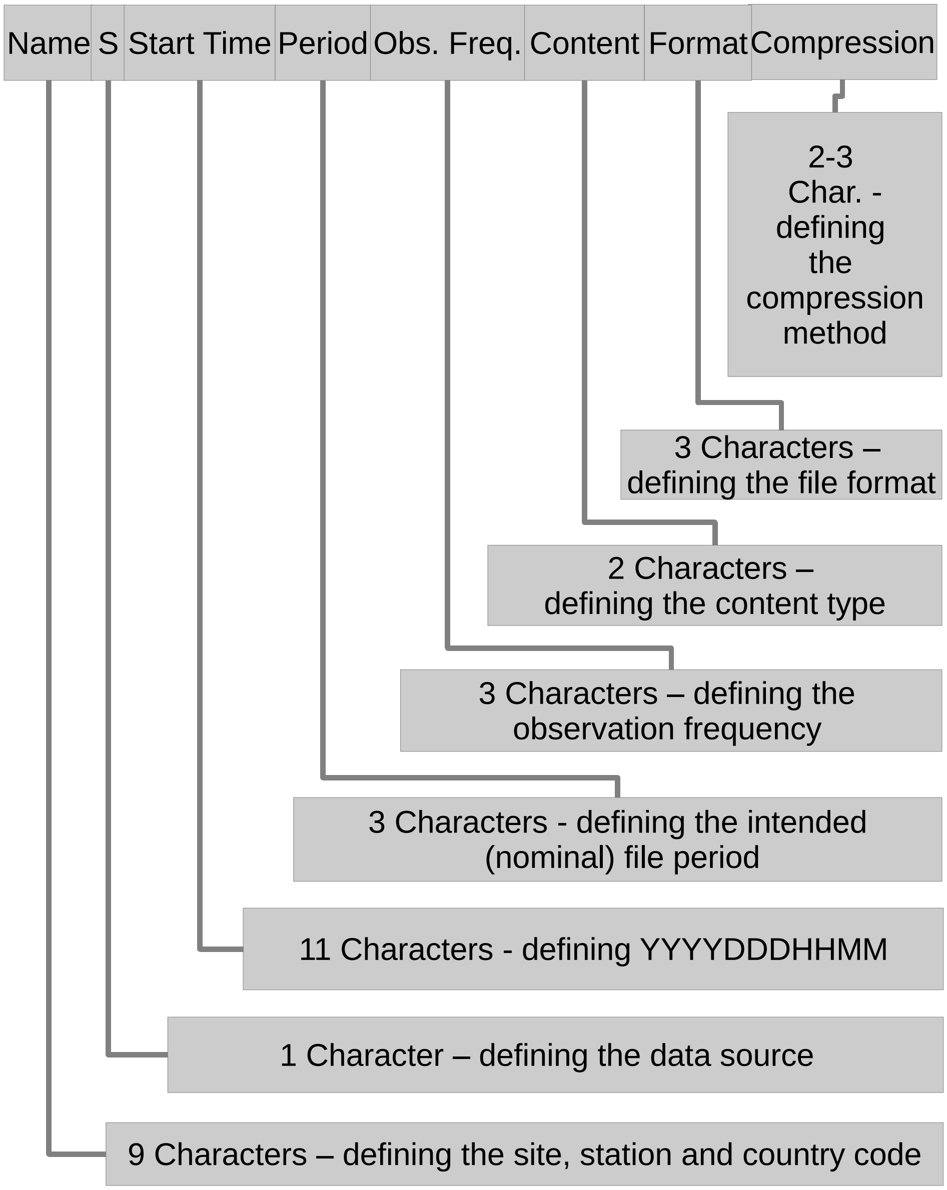

Figure 2: Recommended filename parameters.

All elements are fixed length and are separated by an underscore "_" except for the: file type and compression fields that uses a period "." separator. Fields must be padded with zeros to fill the field width. The file compression field is optional. See Appendix A1 for a detailed description of the RINEX 3.02 file naming convention. Table 1 below lists sample file names for GNSS observation and navigation files.

File Name	Comments
ALGO00CAN_R_20121601000_01H_01S_MO.rnx	Mixed RINEX GNSS observation file containing 1 hour of data, with an observation every second.
ALGO00CAN_R_20121601000_15M_01S_GO.rnx	GPS RINEX observation file containing 15 minutes of data, with an observation every second.
ALGO00CAN_R_20121601000_01H_05Z_MO.rnx	Mixed RINEX GNSS observation file containing 1 hour of data, with 5 observations per second.
ALGO00CAN_R_20121601000_01D_30S_GO.rnx	GPS RINEX observation file containing 1 day of data, with an observation every 30 seconds.
ALGO00CAN_R_20121601000_01D_30S_MO.rnx	Mixed RINEX GNSS observation file containing 1 day of data, with an observation every 30 seconds.
ALGO00CAN_R_20121600000_01D_GN.rnx	RINEX GPS navigation file, containing one days data.
ALGO00CAN_R_20121600000_15M_RN.rnx	RINEX GLONASS navigation file, containing one days data

Table 1: Description of filename parameters

In order to further reduce the size of observation files Yuki Hatanaka developed a compression scheme that takes advantage of the structure of the RINEX observation data by forming higher-order differences in time between observations of the same type and satellite. This compressed file is also an ASCII file that is subsequently compressed again using the above mentioned standard compression programs.

References for the Hatanaka compression scheme: See e.g.

- **http://terras.gsi.go.jp/ja/crx2rnx.html**
- IGSMails 1525,1686,1726,1763,1785,4967,4969,4975

The file naming and compression recommendations are strictly speaking not part of the RINEX format definition. However, they significantly facilitate the exchange of RINEX data in large user communities like IGS.

5. RINEX VERSION 3 FEATURES

The following section contains features that have been introduced for RINEX Version 3:

5.1 Observation codes

The new signal structures for GPS, Galileo and BDS make it possible to generate code and phase observations based on one or a combination of several channels: Two-channel signals are composed of I and Q components, three-channel signals of A, B, and C components. Moreover a wideband tracking of a combined E5a + E5b frequency tracking is possible. In order to keep the observation codes short but still allow for a detailed characterization of the actual signal generation the length of the codes is increased from two (Version 1 and 2) to three by adding a signal generation attribute:

The observation code **tna** consists of three parts:

**t** :observation type	**C** = pseudorange,	**L** = carrier phase,	**D** = doppler,	**S** = signal strength)
**n** :band / frequency	**1, 2,...,8**			
**a** : attribute	tracking mode or channel, e.g., **I**, **Q**, etc			

Examples:

- **L1C:** C/A code-derived L1 carrier phase (GPS, GLONASS) Carrier phase on E2-L1-E1 derived from C channel (Galileo)
- **C2L:** L2C pseudorange derived from the L channel (GPS)

Tables 2 to 7 describe each GNSS constellation and the frequencies and signal encoding methods used.

GNSS System	Freq. Band /Frequency	Channel or Code	Observation Codes			
			Pseudo Range	Carrier Phase	Doppler	Signal Strength
GPS	L1/1575.42	C/A	C1C	L1C	D1C	S1C
		L1C (D)	C1S	L1S	D1S	S1S
		L1C (P)	C1L	L1L	D1L	S1L
		L1C (D+P)	C1X	L1X	D1X	S1X
		P	C1P	L1P	D1P	S1P
		Z-tracking and similar (AS on)	C1W	L1W	D1W	S1W
		Y	C1Y	L1Y	D1Y	S1Y
		M	C1M	L1M	D1M	S1M
		codeless		L1N	D1N	S1N
	L2/1227.60	C/A	C2C	L2C	D2C	S2C
		L1(C/A)+(P2-P1) (semi-codeless)	C2D	L2D	D2D	S2D
		L2C (M)	C2S	L2S	D2S	S2S
		L2C (L)	C2L	L2L	D2L	S2L
		L2C (M+L)	C2X	L2X	D2X	S2X
		P	C2P	L2P	D2P	S2P
		Z-tracking and similar (AS on)	C2W	L2W	D2W	S2W
		Y	C2Y	L2Y	D2Y	S2Y
		M	C2M	L2M	D2M	S2M
		codeless		L2N	D2N	S2N
	L5/1176.45	I	C5I	L5I	D5I	S5I
		Q	C5Q	L5Q	D5Q	S5Q
		I+Q	C5X	L5X	D5X	S5X

Table 2 : RINEX Version 3.02 GPS observation codes

GNSS System	Freq. Band /Frequency	Channel or Code	Observation Codes			
			Pseudo Range	Carrier Phase	Doppler	Signal Strength
GLONASS	G1/ 1602+k*9/16 k= -7….+12	C/A	C1C	L1C	D1C	S1C
		P	C1P	L1P	D1P	S1P
	G2/ 1246+k*716	C/A (GLONASS M)	C2C	L2C	D2C	S2C
		P	C2P	L2P	D2P	S2P
	G3 / 1202.025	I	C3I	L3I	D3I	S3I
		Q	C3Q	L3Q	D3Q	S3Q
		I+Q	C3X	L3X	D3X	S3X

Table 3 : RINEX Version 3.02 GLONASS observation codes

GNSS System	Freq. Band /Frequency	Channel or Code	Observation Codes			
			Pseudo Range	Carrier Phase	Doppler	Signal Strength
Galileo	E1 / 1575.42	A PRS	C1A	L1A	D1A	S1A
		B I/NAV OS/CS/SoL	C1B	L1B	D1B	S1B
		C no data	C1C	L1C	D1C	S1C
		B+C	C1X	L1X	D1X	S1X
		A+B+C	C1Z	L1Z	D1Z	S1Z
	E5a / 1176.45	I F/NAV OS	C5I	L5I	D5I	S5I
		Q no data	C5Q	L5Q	D5Q	S5Q
		I+Q	C5X	L5X	D5X	S5X
	E5b / 1207.140	I I/NAV OS/CS/SoL	C7I	L7I	D7I	S7I
		Q no data	C7Q	L7Q	D7Q	S7Q
		I+Q	C7X	L7X	D7X	S7X
	E5(E5a+E5b) / 1191.795	I	C8I	L8I	D8I	S8I
		Q	C8Q	L8Q	D8Q	S8Q
		I+Q	C8X	L8X	D8X	S8X
	E6 / 1278.75	A PRS	C6A	L6A	D6A	S6A
		B C/NAV CS	C6B	L6B	D6B	S6B
		C no data	C6C	L6C	D6C	S6C
		B+C	C6X	L6X	D6X	S6X
		A+B+C	C6Z	L6Z	D6Z	S6Z

Table 4 : RINEX Version 3.02 Galileo observation codes

GNSS System	Freq. Band/ Frequency	Channel or Code	Observation Codes			
			Pseudo Range	Carrier Phase	Doppler	Signal Strength
**SBAS**	L1 / 1575.42	C/A	C1C	L1C	D1C	S1C
	L5 / 1176.45	I	C5I	L5I	D5I	S5I
		Q	C5Q	L5Q	D5Q	S5Q
		I+Q	C5X	L5X	D5X	S5X

Table 5 : RINEX Version 3.02 SBAS observation codes

GNSS System	Freq. Band / Frequency	Channel or Code	Observation Codes			
			Pseudo Range	Carrie r Phase	Doppler	Signal Strength
**QZSS**	L1 / 1575.42	C/A	C1C	L1C	D1C	S1C
		L1C (D)	C1S	L1S	D1S	S1S
		L1C (P)	C1L	L1L	D1L	S1L
		L1C (D+P)	C1X	L1X	D1X	S1X
		L1-SAIF	C1Z	L1Z	D1Z	S1Z
	L2 / 1227.60	L2C (M)	C2S	L2S	D2S	S2S
		L2C (L)	C2L	L2L	D2L	S2L
		L2C (M+L)	C2X	L2X	D2X	S2X
	L5 / 1176.45	I	C5I	L5I	D5I	S5I
		Q	C5Q	L5Q	D5Q	S5Q
		I+Q	C5X	L5X	D5X	S5X
	LEX(6) / 1278.75	S	C6S	L6S	D6S	S6S
		L	C6L	L6L	D6L	S6L
		S+L	C6X	L6X	D6X	S6X

Table 6 : RINEX Version 3.02 QZSS observation codes

GNSS System	Freq. Band / Frequency	Channel or Code	Observation Codes			
			Pseudo Range	Carrier Phase	Doppler	Signal Strength
BDS	B1 / 1561.098	I	C1I	L1I	D1I	S1I
		Q	C1Q	L1Q	D1Q	S1Q
		I+Q	C1X	L1X	D1X	S1X
	B2 / 1207.14	I	C7I	L7I	D7I	S7I
		Q	C7Q	L7Q	D7Q	S7Q
		I+Q	C7X	L7X	D7X	S7X
	B3 / 1268.52	I	C6I	L6I	D6I	S6I
		Q	C6Q	L6Q	D6Q	S6Q
		I+Q	C6X	L6X	D6X	S6X

Table 7 : RINEX Version 3.02 BDS observation codes

For Galileo the band/frequency number **n** does not necessarily agree with the official frequency numbers: **n** = 7 for E5b, **n** = 8 for E5a+b.

GPS-SBAS and –pseudorandom noise (PRN) code assignments:
See e.g., http://www.losangeles.af.mil/library/factsheets/factsheet.aspid=8618

Antispoofing (AS) of GPS: True codeless GPS receivers (squaring-type receivers) use the attribute **N**. Semi-codeless receivers tracking the first frequency using C/A code and the second frequency using some codeless options use attribute **D**. Z-tracking under AS or similar techniques to recover pseudorange and phase on the "P-code" band use attribute **W**. Y-code tracking receivers use attribute **Y**.

Appendix Table A19 enumerates the fractional phase corrections required to align each signal to the frequencies reference signal.

As all observations affected by "AS on" get now their own attribute (codeless, semi-codeless, Z-tracking and similar) the Antispoofing flag introduced into the observation data records of RINEX Version 2 has become obsolete.

5.2 Satellite system-dependent list of observables

The order of the observations stored per epoch and satellite in the observation records is given by a list of observation codes in a header record. As the types of the observations actually generated by a receiver may heavily depend on the satellite system RINEX Version 3 requests system-dependent observation code list (header record type **SYS / # / OBS TYPES**).

5.3 Marker type

In order to indicate the nature of the marker a **MARKER TYPE** header record has been defined:

Marker Type	Description
Geodetic	Earth-fixed high-precision monument
Non Geodetic	Earth-fixed low-precision monument
Non_Physical	Generated from network processing
Space borne	Orbiting space vehicle
Air borne	Aircraft, balloon, etc.
Water Craft	Mobile water craft
Ground Craft	Mobile terrestrial vehicle
Fixed Buoy	"Fixed" on water surface
Floating Buoy	Floating on water surface
Floating Ice	Floating ice sheet, etc
Glacier	"Fixed" on a glacier
Ballistic	Rockets, shells, etc
Animal	Animal carrying a receiver
Human	Human being

Table 8: Proposed marker type keywords

The record is required except for **GEODETIC** and **NON_GEODETIC** marker types.

Attributes other than **GEODETIC** and **NON_GEODETIC** will tell the user program that the data were collected by a moving receiver. The inclusion of a "start moving antenna" record (event flag 2) into the data body of the RINEX file is therefore not necessary. Event flags 2 and 3 are still necessary to flag alternating kinematic and static phases of a receiver visiting multiple earth-fixed monuments, however.

Users may define other project-dependent keywords.

5.4 Half-wavelength observations, half-cycle ambiguities

Half-wavelength observations (collected by **codeless** squaring techniques) get their own observation codes. A special wavelength factor header line and bit 1 of the LLI flag in the observation records are not necessary anymore. If a receiver changed between squaring and full cycle tracking within the time period of a RINEX file, observation codes for both types of observations have to be inserted into the respective **SYS / # / OBS TYPES** header record.
Half-wavelength phase observations are stored in full cycles. Ambiguity resolution however has to account for half wavelengths!

Full-cycle observations collected by receivers with possible half cycle ambiguity (e.g., during acquisition or after loss of lock) are to be flagged with Loss of Lock Indicator bit 1 set (see Appendix Table A3).

5.5 Scale factor

The *optional* **SYS / SCALE FACTOR** record allows e.g., to store phase data with 0.0001 cycles resolution if the data was multiplied by a scale factor of 10 before being stored into RINEX file. Used to increase resolution by 10, 100, etc only. It is a modification of the Version 2.20 **OBS SCALE FACTOR** record.

5.6 Information about receivers on a vehicle

For the processing of data collected by receivers on a vehicle the following additional information can be provided by special header records:

- Antenna position (position of the antenna reference point) in a body-fixed coordinate system: **ANTENNA: DELTA X/Y/Z**
- Bore-sight of antenna: The unit vector of the direction of the antenna axis towards the GNSS satellites. It corresponds to the vertical axis on earth-bound antenna: **ANTENNA: B.SIGHT XYZ**
- Antenna orientation: Zero-direction of the antenna. Used for the application of "azimuth"-dependent phase center variation models (see 6.14 below): **ANTENNA: ZERODIR XYZ**
- Current center of mass of the vehicle (for spaceborne receivers): **CENTER OF MASS: XYZ**
- Average phase center position: **ANTENNA: PHASECENTER (**see below)

All three quantities have to be given in the same body-fixed coordinate system. The attitude of the vehicle has to be provided by separate attitude files in the same body-fixed coordinate system.

5.7 Signal strengths

The generation of the RINEX signal strength indicators sn_rnx in the data records (1 = very weak,…,9 = very strong) are standardized in case the raw signal strength[1] sn_raw is given in **dbHz**:

```
sn_rnx  =  MIN(MAX(INT(sn_raw/6),1),9)
```

Signal to Noise ratio(dbHz)	Signal to Noise ratio(RINEX)
< 12	1
12-17	2
18-23	3
24-29	4
30-35	5
36-41	6
42-47	7
48-53	8
≥ 54	9

Table 9: Standardized S/N indicators

The raw carrier to noise ratio can be optionally stored as **`Sna`** observations in the data records should be stored in dbHz if possible. The new **`SIGNAL STRENGTH UNIT`** header record can be used to indicate the units of these observations.

5.8 Date/time format in the PGM / RUN BY / DATE header record

The format of the generation time of the RINEX files stored in the second header record **`PGM / RUN BY / DATE`** is now defined to be

`yyyymmdd hhmmss zone`

`zone:` 3 – 4 character code for the time zone

It is recommended to use **`UTC`** as time zone. Set **`zone`** to **`LCL`** if local time was used with unknown local time system code.

[1] S/N is the raw S/N at the output of the correlators, without attempting to recover any correlation losses

5.9 Antenna phase center header record

An *optional* header record for antenna phase center positions **ANTENNA: PHASECENTER** is defined to allow for higher precision positioning without need of additional external antenna information. It can be useful in well-defined networks or applications. It contains the position of an *average* phase center relative to the antenna reference point (ARP) for a specific frequency and satellite system. On vehicles the phase center position can be reported in the body-fixed coordinate system (**ANTENNA: DELTA X/Y/Z**). See 6.14 below. Regarding the use of phase center variation corrections see 5.15.

5.10 Antenna orientation

Header records have been defined to report the orientation of the antenna zero-direction as well as the direction of its vertical axis (bore-sight) if mounted tilted on a fixed station. The header records can also be used for antennas on vehicles. See 6.14 below.

5.11 Observation data records

Apart from the new observation code definitions the most conspicuous modification of the RINEX format concerns the observation records. As the types of the observations and their order within a data record depend on the satellite system, the new format should make it easier for programs as well as human beings to read the data records. Each observation record begins with the satellite number **snn**, the epoch record starts with special character **>**. It is now also much easier to synchronize the reading program with the next epoch record in case of a corrupted data file or when streaming observation data in a RINEX-like format. The record length limitation to 80 characters of RINEX Versions 1 and 2 ***has been removed***.

For the following list of observation types for the four satellite systems **G,S,E,R :**

```
G    5 C1P L1P L2C C2C S2C                                    SYS / # / OBS TYPES
R    2 C1C L1C                                                SYS / # / OBS TYPES
E    2 L1B L5I                                                SYS / # / OBS TYPES
S    2 C1C L1C                                                SYS / # / OBS TYPES
```

Table 10: Example for a list of observation types

The epoch and observation records look as follows:

```
> 2006 03 24 13 10 54.0000000  0  7      -0.123456789210
G06  23619095.450      -53875.632 8    -41981.375 5  23619112.008          24.158
G09  20886075.667      -28688.027 9    -22354.535 6  20886082.101          38.543
G12  20611072.689       18247.789 9     14219.770 8  20611078.410          32.326
R21  21345678.576       12345.567 5
R22  22123456.789       23456.789 5
E11     65432.123 5     48861.586 7
S20  38137559.506      335849.135 9
```

Table 11: Example for observation data records

The receiver clock correction in the epoch record has been placed such that it could be preceded

by an identifier to make it system-dependent in a later format revision, if necessary. The clock correction is optional and is given in units of seconds.

5.12 Ionosphere delay as pseudo-observables

RINEX files could also be used to make available additional information linked to the actual observations. One such element is the ionosphere delay having been determined or derived from an ionosphere model. We add the ionosphere phase delay expressed in full cycles of the respective satellite system-dependent wavelength as pseudo-observable to the list of the RINEX observables.

**T**: observation type	**I =** Ionosphere phase delay
**n**: band/frequency	**1, 2, ...,8**
**a**: attribute	blank

The ionosphere pseudo-observable has to be included into the list of observables of the respective satellite system. Only one ionosphere observable per satellite is allowed.

The user adds the ionosphere delay to the raw phase observation of the same wavelength and converts it to other wavelengths and to pseudorange corrections in meters:

corr_phase(fi)	=	raw_phase(fi)	+	d_ion(fi)
corr_prange(fi)	=	raw_prange(fi)	-	d_ion(fi) • c/fi
d_ion(fk)	=	d_ion(fi)	•	(fi/fk)**2 (accounting for 1st order effects only)

d_ion(fi): Given ionospheric phase correction for frequency fi

5.13 Channel numbers as pseudo-observables

For special applications it might be necessary to know the receiver channel numbers having been assigned by the receiver to the individual satellites. We may include this information as another pseudo-observable:

- **t** : observation type: **X** = Receiver channel number
- **n** : band / frequency : **1**
- **a** : attribute: blank

Lowest channel number allowed is 1 (re-number channels beforehand, if necessary). In case of a receiver using multiple channels for one satellite the channels could be packed with two digits each right-justified into the same data field, order corresponding to the order of the observables concerned. Format `F14.3` according to `(<5-nc>(2X),<nc>I2.2,'.000')`, `nc` being the number of channels.

Restriction: Not more than 5 channels and channel numbers <100.

Examples:

- **`0910.000`** for channels 9 and 10
- **`010203.000`** for channels 1, 2, and 3

```
-----F14.3----
```

5.14 Corrections of differential code biases (DCBs)

For special high-precision applications it might be useful to generate RINEX files with corrections of the differential code biases (DCBs) already applied. There are programs available to correct the observations in RINEX files for differential code biases (e.g., **cc2noncc**, J. Ray 2005). This can be reported by special header records **`SYS / DCBS APPLIED`** pointing to the file containing the applied corrections.

5.15 Corrections of antenna phase center variations (PCVs)

For more precise applications an elevation-or elevation and azimuth-dependent phase center variation (pcv) model for the antenna (referring to the agreed-upon ARP) should be used. For special applications it might be useful to generate RINEX files with these variations already applied. This can be reported by special header records **`SYS / PCVS APPLIED`** pointing to the file containing the PCV correction models.

5.16 Navigation message files

The header portion has been unified (with respect to the format definitions) for all satellite systems. The data portion contains now in the first record of each message block in addition to the satellite number also the code for the satellite system.

```
G06 1999 09 02 17 51 44 -.839701388031D-03 -.165982783074D-10  .000000000000D+00
```

Header records with system-dependent contents also contain the system identifier. They are repeated for each system, if applicable.

```
GPSA    .1676D-07   .2235D-07   .1192D-06   .1192D-06       IONOSPHERIC CORR
GPSB    .1208D+06   .1310D+06  -.1310D+06  -.1966D+06       IONOSPHERIC CORR
GAL     .1234D+05   .2345D+04  -.3456D+03                   IONOSPHERIC CORR
```

6. ADDITIONAL HINTS AND TIPS

6.1 Versions

Programs developed to read RINEX files have to verify the version number. Files of newer versions may look different even if they do not use any of the newer features

6.2 Leading blanks in CHARACTER fields

We propose that routines to read files automatically delete leading blanks in any CHARACTER input field. Routines creating RINEX files should also left-justify all variables in the CHARACTER fields.

6.3 Variable-length records

ASCII files usually have variable record lengths, so we recommend to first read each observation record into a blank string long enough to accommodate the largest possible observation record[2] and decode the data afterwards. In variable length records, empty data fields at the end of a record may be missing, especially in the case of the optional receiver clock offset.

6.4 Blank fields

In view of future modifications we recommend to carefully skip any fields currently defined to be blank (format fields nX), because they may be assigned to new contents in future versions.

6.5 Order of the header records, order of data records

As the header record descriptors in columns 61-80 are mandatory, the programs reading a RINEX Version 3 header must decode the header records with formats according to the record descriptor, provided the records have been first read into an internal buffer.

We therefore propose to allow free ordering of the header records, with the following exceptions:

- The **RINEX VERSION / TYPE** record must be the first record in a file
- The **SYS / # / OBS TYPES** record(s) should precede any **SYS / DCBS APPLIED** and **SYS / SCALE FACTOR** records.
- The **# OF SATELLITES** record (if present) should be immediately followed by the corresponding number of **PRN / # OF OBS** records. (These records may be handy for documentary purposes. However, since they may only be created after having read whole raw data file we define them to be optional.
- The **END OF HEADER** of course is the last record in the header

---

[2] Defined by the satellite system with the largest number of possible observables plus any "pseudo-observables" like ionosphere, etc. The length limitation to 80 characters of RINEX Versions 1 and 2 has been removed.

Data records: Multiple epoch data records with identical time tags are not allowed (exception: Event records). Epochs have to appear ordered in time.

6.6 Missing items, duration of the validity of values

Items that are not known at the file creation time can be set to zero or blank or the respective record may be completely omitted. Consequently items of missing header records will be set to zero or blank by the program reading RINEX files. Trailing blanks may be truncated from the record.

Each value remains valid until changed by an additional header record.

6.7 Unknown / Undefined observation types and header records

It is a good practice for a program reading RINEX files to make sure that it properly deals with unknown observation types, header records or event flags by skipping them and/or reporting them to the user. The program should also check the RINEX version number in the header record and take proper action if it cannot deal with it.

6.8 Event flag records

The "number of satellites" also corresponds to the number of records of the same epoch following the ***EPOCH*** record. Therefore it may be used to skip the appropriate number of data records if certain event flags are not to be evaluated in detail.

6.9 Receiver clock offset

A receiver-derived clock offset can optionally be reported in the RINEX observation files. In order to remove uncertainties about whether the data (epoch, pseudorange, phase) have been corrected or not by the reported clock offset, RINEX Versions 2.10 onward requests a clarifying header record: **RCV CLOCK OFFS APPL**. It would then be possible to reconstruct the original observations, if necessary.

6.10 Two-digit years

RINEX version 2 stores the years of data records with two digits only. The header of observation files contains a **TIME OF FIRST OBS** record with the full four-digit year, the GPS nav messages contain the GPS week numbers. From these two data items the unambiguous year can easily be reconstructed.

A hundred-year ambiguity occurs in the met data and GLONASS and GEO nav messages: Instead of introducing a new **TIME OF FIRST OBS** header line it is safe to stipulate that any two-digit years in RINEX Version 1 and Version 2.xx files are understood to represent

80-99: 1980-1999
00-79: 2000-2079

Full 4-digit year fields are/will be defined in the RINEX version 3 files.

6.11 Fit interval (GPS navigation message file)

Bit 17 in word 10 of subframe 2 is a “fit interval” flag which indicates the curve-fit interval used by the GPS Control Segment in determining the ephemeris parameters, as follows (see ICD-GPS-200, 20.3.3.4.3.1):

0 = 4 hours
1 = greater than 4 hours.

Together with the IODC values and Table 20-XII the actual fit interval can be determined. The second value in the last record of each message shall contain the fit interval in hours determined using IODC, fit flag, and Table 20-XII, according to the Interface Document ICD-GPS-200.

6.12 Satellite health (GPS navigation message file)

The health of the signal components (bits 18 to 22 of word three in subframe one) are included from version 2.10 on using the health value reported in the second field of the sixth navigation message record.

A program reading RINEX files could easily decide if bit 17 only or all bits (17-22) have been written:

RINEX Value:	0	Health OK
RINEX Value:	1	Health not OK (bits 18-22 not stored)
RINEX Value:	>32	Health not OK (bits 18-22 stored)

6.13 Transmission time of message (GPS navigation message file)

The transmission time of a message can be shortly before midnight Saturday/Sunday, the ToE and ToC of the message already in the next week.

As the reported week in the RINEX nav message (***BROADCAST ORBIT -5*** record) goes with ToE (this is different from the GPS week in the original satellite message!), the transmission time of message should be reduced by 604800 (i.e., will become negative) to also refer to the same week.

6.14 Antenna references, phase centers

We distinguish between

- The ***marker***, i.e. the geodetic reference monument, on which an antenna is mounted directly with forced centering or on a tripod.
- The ***antenna reference point*** (ARP), i.e., a well-defined point on the antenna, e.g., the center of the bottom surface of the preamplifier. The antenna height is measured from the marker to the ARP and reported in the **ANTENNA: DELTA H/E/N** header record. Small horizontal eccentricities of the ARP w/r to the marker can be reported in the same record. On vehicles the position of the ARP is reported in the body-fixed coordinate system in an **ANTENNA: DELTA X/Y/Z** header record.
- The ***average phase center***: A frequency-dependent and minimum elevation-angle-dependent position of the average phase center above the antenna reference point. Its position is important to know in mixed-antenna networks. It can be given in an absolute sense or relative to a reference antenna using the optional header record: **ANTENNA: PHASECENTER**. For fixed stations the components are in north/east/up direction, on vehicles the position is reported in the body-fixed system X,Y,Z. For more precise applications an elevation-dependent or elevation and azimuth-dependent phase center variation (PCV) model for the antenna (referring to the agreed-upon ARP) should be

used. For special applications it might be useful to generate RINEX files with these corrections already applied. This can be reported by special header records **SYS / PCVS APPLIED** pointing to the file containing the PCV correction models.

- The ***orientation*** of the antenna: The “zero direction” is usually oriented towards north on fixed stations. Deviations from the north direction can be reported with the azimuth of the zero-direction in an **ANTENNA: ZERODIR AZI** header record. On vehicles the zero-direction is reported as a unit vector in the body-fixed coordinate system in an **ANTENNA: ZERODIR XYZ** header record. The zero direction of a tilted antenna on a fixed station can be reported as unit vector in the left-handed north/east/up local coordinate system in an **ANTENNA: ZERODIR XYZ** header record.

- The ***bore-sight direction*** of an antenna on a vehicle: The “vertical” symmetry axis of the antenna pointing towards the GNSS satellites. It can be reported as unit vector in the body-fixed coordinate system in the **ANTENNA: B.SIGHT XYZ** record. A tilted antenna on a fixed station could be reported as unit vector in the left-handed north/east/up local coordinate system in the same type of header record.

To be able to interpret the various positions correctly it is important that the **MARKER TYPE** record is included in the RINEX header.

7. RINEX UNDER ANTISPOOFING (AS)

Some receivers generate code (pseudorange) delay differences between the first and second frequency using cross-correlation techniques when AS is on and may recover the phase observations on L2 in full cycles. Using the C/A code delay on L1 and the observed difference it is possible to generate a code delay observation for the second frequency. Other receivers recover P code observations by breaking down the Y code into P and W code.

Most of these observations may suffer from an increased noise level. In order to enable the post-processing programs to take special actions, such AS-infected observations have been flagged in RINEX Version 2 using bit number 2 of the Loss of Lock Indicators (i.e. their current values are increased by 4). In Version 3 there are special attributes for the observation type to more precisely characterize the observable (codeless, semi-codeless, Z-tracking or similar techniques when AS on, L2C, P-code when AS off, Y-code tracking), making the AS flag obsolete.

8. DEALING WITH DIFFERENT SATELLITE SYSTEMS

8.1 Time system identifier

GPS time runs, apart from small differences (<< 1 microsecond), parallel to UTC. But it is a continuous time scale, i.e. it does not insert any leap seconds. GPS time is usually expressed in GPS weeks and GPS seconds past 00:00:00 (midnight) Saturday/Sunday. GPS time started with week zero at 00:00:00 UT (midnight) on January 6, 1980. Between 1980 and 2012 16 leap seconds have been introduced into UTC.

The GPS week is transmitted by the satellites as a 10 bit number. It has a roll-over after week 1023. The first roll.-over happened on August 22, 1999, 00:00:00 GPS time.

In order to avoid ambiguities the GPS week reported in the RINEX navigation message files is a continuous number without roll-over, i.e. …1023, 1024, 1025, …

We use **`GPS`** as time system identifier for the reported GPS time.

QZSS runs on QZSS time, which conforms to UTC Japan Standard Time Group (JSTG) time and the offset with respect to GPS time is controlled. The following properties apply to the QZSS time definition: the length of one second is defined with respect to International Atomic Time (TAI); QZSS time is aligned with GPS time (offset from TAI by integer seconds); the QZSS week number is defined with respect to the GPS week.

We use **`QZS`** as a time system identifier for the reported QZSS time

GLONASS is basically running on UTC (or, more precisely, GLONASS system time linked to UTC(SU)), i.e. the time tags are given in UTC and not GPS time. It is not a continuous time, i.e. it introduces the same leap seconds as UTC. The reported GLONASS time has the same hours as UTC and not UTC+3 h as the original GLONASS System Time!

We use **`GLO`** as time system identifier for the reported GLONASS time.

Galileo runs on Galileo System Time (GST), which is, apart from small differences (tens of nanoseconds), nearly identical to GPS time:

- The Galileo week starts at midnight Saturday/Sunday at the same second as the GPS week.
- The GST week as transmitted by the satellites is a 12 bit value with a roll-over after week 4095. The GST week started at zero at the first roll-over of the broadcast GPS week after 1023, i.e. at Sun, 22-Aug-1999 00:00:00 GPS time

In order to remove possible misunderstandings and ambiguities the Galileo week reported in the RINEX navigation message files is a continuous number without roll-over, i.e., …4095,4096,4097,… and *it is aligned to the GPS week.*

We use **`GAL`** as time system identifier for this reported Galileo time.

The **BDS** Time (BDT) System is a continuous timekeeping system, with its length of second being a SI second. BDT zero time started at 00:00:00 UTC on January 1st, 2006 (GPS week 1356). BDT is synchronized with UTC within 100 nanoseconds (modulo 1 second).

- The **BDT** week starts at midnight Saturday/Sunday.
- The **BDT** week is transmitted by the satellites as a 13 bit number. It has a roll-over after week 8191. In order to avoid ambiguities the BDT week reported in the RINEX navigation message files is a continuous number without roll-over, i.e. …8191, 8192, 8193, …

We use **BDT** as time system identifier for the reported BDS time.

Constellation /Archival Time Representation	GPS Ephemeris Week Period #1	GPS Ephemeris Week Period #2	GPS Ephemeris Week Period #3	GPS Ephemeris Week Period #4	GPS Ephemeris Week Period #5	GPS Ephemeris Week Period #6
**GPS Broadcast**	0 – 1023	0 – 1023	0 – 1023	0 – 1023	0 – 1023	0 – 1023
**QZSS Broadcast**		0 – 1023	0 – 1023	0 – 1023	0 – 1023	0 – 1023
**GPS/QZSS RINEX**	0 – 1023	1024 – 2047	2048 – 3071	3072 – 4095	4096 – 5119	5120 -6143
**GST**		0 – 1023	1024 – 2047	2048 – 3071	3072 – 4095	0 – 1023
**GAL**		1024 – 2047	2048 – 3071	3072 – 4095	4096 – 5119	5120 – 6143
**BDS**		0(1356) – 691	692 – 1715	1716 – 2739	2740 – 3763	3764 – 4787

Table 12: Relations between GPS, QZSS, GST, GAL and BDS weeks

The header records **`TIME OF FIRST OBS`** and (if present) **`TIME OF LAST OBS`** in pure GPS, GLONASS, Galileo, QZSS or BDS observation files **can**, in mixed

GPS/GLONASS/Galileo/QZSS/BDS observation files **must** contain the time system identifier defining the system that all time tags in the file are referring to:

- **GPS** to identify GPS time,
- **GLO** to identify the GLONASS UTC time system
- **GAL** to identify Galileo time.
- **QZS** to identify QZSS time.
- **BDT** to identify BDS time.

Pure GPS observation files default to **GPS**, pure GLONASS files default to **GLO**, pure Galileo files default to **GAL** and similarly pure BDS observation files default to BDT.

Apart from the small errors in the realizations of the different time systems, the relations between the sys-terms are:

GLO	=	UTC	=	GPS	-	ΔtLS
GPS	=	GAL	=	UTC	+	ΔtLS

GPS	=	BDT	+	ΔtLS(2006)

Where :

ΔtLS	=	Delta time between GPS and UTC due to leap seconds, as transmitted by the GPS satellites in the almanac (2005: ΔtLS = 13, 2006: ΔtLS = 14, 2008: ΔtLS = 15 and 2012: ΔtLS = 16).

In order to have the current number of leap seconds available we recommend to include ΔtLS by a **LEAP SECOND** line into the RINEX file headers.

If there are known non-integer biases between “GPS receiver clock”, “GLONASS receiver clock” or “Galileo receiver clock” in the same receiver, they should be applied in the process of RINEX conversion. In this case the respective code and phase observations have to be corrected too (c * bias if expressed in meters).

Unknown biases will have to be solved for during the post processing

The small differences (modulo 1 second) between Galileo system time, GLONASS system time, UTC(SU), UTC(USNO) and GPS system time have to be dealt with during the post-processing and not before the RINEX conversion. It may also be necessary to solve for remaining differences during the post-processing.

8.2 Pseudorange definition

The pseudorange (code) measurement is defined to be equivalent to the difference of the time of reception (expressed in the time frame of the receiver) and the time of transmission (expressed in the time frame of the satellite) of a distinct satellite signal.

In a mixed-mode GPS/GLONASS/Galileo/QZSS/BDS receiver referring all pseudorange observations to one receiver clock only,

- the raw GLONASS pseudoranges will show the current number of leap seconds between GPS/GAL/BDT time and GLONASS time if the receiver clock is running in the GPS, GAL or BDT time frame
- the raw GPS, Galileo and BDS pseudoranges will show the negative number of leap seconds between GPS/GAL/BDT time and GLONASS time if the receiver clock is running in the GLONASS time frame

In order to avoid misunderstandings and to keep the code observations within the format fields, the pseudo-ranges must be corrected in this case as follows:

PR_mod(GPS)	=	PR(GPS)	+	C* ΔtLS	if generated with a receiver clock running in the GLONASS time frame
PR_mod(GAL)	=	PR(GAL)	+	C* ΔtLS	if generated with a receiver clock running in the GLONASS time frame
PR_mod(BDT)	=	PR(BDT)	+	C* ΔtLS	if generated with a receiver clock running in the GLONASS time frame
PR_mod(GLO)	=	PR(GLO)	-	C* ΔtLS	if generated with a receiver clock running in the GPS or GAL time frame

to remove the contributions of the leap seconds from the pseudoranges.

ΔtLS is the actual number of leap seconds between GPS/GAL and GLO time, as broadcast in the GPS almanac and distributed in Circular T of BIPM.

8.3 RINEX navigation message files

The header section of the RINEX version 3.00 navigation message files have been slightly changed compared to the previous version 2. The format of the header section is identical for all satellite systems, i.e., GPS, GLONASS, Galileo, SBAS, QZSS and BDS.

The data portion of the navigation message files contains records with floating point numbers. The format is identical for all satellite systems, the number of records per message and the contents, however, are satellite system-dependent. The format of the version 3 data records has been changed slightly, the satellite codes now contain also the satellite system identifier.

It is possible to generate mixed navigation message files, i.e. files containing navigation messages of more than one satellite system. Header records with system-dependent contents have to be repeated for each satellite system, if applicable. Using the satellite system identifier of the satellite code the reading program can determine the number of data records to be read for each message block.

The time tags of the navigation messages (e.g., time of ephemeris, time of clock) are given in the respective satellite time systems!

It is recommended to avoid storing redundant navigation messages (e.g., the same message broadcast at different times) into the RINEX file.

8.3.1 RINEX navigation message files for GLONASS

The header section and the first data record (epoch, satellite clock information) are equal to the GPS navigation file. The following three records contain the satellite position, velocity and acceleration, the clock and frequency biases as well as auxiliary information as health, satellite frequency (channel), age of the information.

The corrections of the satellite time to UTC are as follows:

GPS: Tutc = Tsv –af0 –af1 *(Tsv-Toc) -... –A0 -... –ΔtLS
GLONASS: Tutc = Tsv + TauN –GammaN*(Tsv-Tb) + TauC

In order to use the same sign conventions for the GLONASS corrections as in the GPS navigation files, the broadcast GLONASS values are stored as:

-TauN, +GammaN, -TauC.

The time tags in the GLONASS navigation files are given in UTC (i.e. **not** Moscow time or GPS time). File naming convention: See above.

8.3.2 RINEX navigation message files for Galileo

The Galileo Open Service allows access to two navigation message types: F/NAV (Freely Accessible Navigation) and I/NAV (Integrity Navigation). The contents of the two messages differs in various items, however, in general it is very similar to the contents of the GPS navigation, e.g. the orbit parameterization is the same. The data blocks of the Galileo RINEX navigation messages are identical to a large extent.

There are items in the navigation message that depend on the origin of the message (F/NAV or I/NAV): The SV clock parameters actually define the satellite clock for the dual-frequency ionosphere-free linear combination. F/NAV reports the clock parameters valid for the E5a-E1 combination, the I/NAV reports the parameters for the E5b-E1 combination. The second parameter in the `Broadcast Orbit 5` record (bits 8 and 9) indicate the frequency pair the

stored clock corrections are valid for.

Some parameters contain the information stored bitwise. The interpretation is as follows:

- Convert the floating point number read from the RINEX file into the nearest integer
- Extract the values of the requested bits from the integer

Example:

`0.170000000000D+02 -> 17 =` 2^4+2^0 `->` Bits 4 and 0 are set, all others are zero

As mentioned above, the GAL week in the RINEX navigation message files is a continuous number; it has been aligned to the GPS week by the program creating the RINEX file.

8.3.3 RINEX navigation message files for GEO satellites

As the GEO broadcast orbit format differs from the GPS message a special GEO navigation message file format has been defined which is nearly identical with the GLONASS navigation message file format.

The header section contains information about the generating program, comments, and the difference between the GEO system time and UTC.

The first data record contains the epoch and satellite clock information, the following records contain the satellite position, velocity and acceleration and auxiliary information (health, URA and IODN).

The time tags in the GEO navigation files are given in the GPS time frame, i.e. **not** UTC.

The corrections of the satellite time to UTC are as follows:

GEO: Tutc = Tsv –aGf0 –aGf1 *(Tsv-Toe) –W0 –ΔtLS

W0 being the correction to transform the GEO system time to UTC. Toe, aGf0, aGf1 see below in the format definition tables.

The *Transmission Time of Message* (**`PRN / EPOCH / SV CLK`** header record) is expressed in GPS seconds of the week. It marks the beginning of the message transmission. It has to refer to the same GPS week as the *Epoch of Ephemerides*. It has to be adjusted by –or + 604800 seconds, if necessary (which would make it lower than zero or larger than 604800, respectively). It is a redefinition of the Version 2.10 *Message frame time*.

Health shall be defined as follows:

- bits 0 to 3 equal to *health* in Message Type 17 (MT17)
- bit 4 is set to 1 if MT17 health is unavailable
- bit 5 is set to 1 if the URA index is equal to 15

8.3.4 RINEX navigation message files for QZSS L1-SAIF

As the QZSS L1-SAIF broadcast orbit format differs from the GPS message a special L1-SAIF navigation message file format has been defined which is nearly identical with the GEO navigation message file format.

The header section contains information about the generating program, comments, and the difference between the L1-SAIF system time and UTC.

The first data record contains the epoch and satellite clock information, the following records contain the satellite position, velocity and acceleration and auxiliary information such as health, age of the data, etc. To compute L1-SAIF satellite position, note that acceleration in navigation message represents only perturbation term and it is necessary to add :

The time tags in the L1-SAIF navigation files are given in the GPS time frame, i.e. **not** UTC.

The corrections of the satellite time to UTC are as follows:

SAIF: Tutc = Tsv –aGf0 –aGf1 *(Tsv-Toe) –W0 –ΔtLS

W0 being the correction to transform the L1-SAIF system time to UTC. Toe, aGf0, aGf1 see below in the format definition tables.

The *Transmission Time of Message* (`PRN / EPOCH / SV CLK` header record) is expressed in GPS seconds of the week. It marks the beginning of the message transmission. It has to refer to the same GPS week as the *Epoch of Ephemerides*. It has to be adjusted by –or + 604800 seconds, if necessary (which would make it lower than zero or larger than 604800, respectively).

Health shall be defined as follows:

- bits 0 to 3 equal to *health* in Message Type 17 (MT17)
- bit 4 is set to 1 if MT17 health is unavailable
- bit 5 is set to 1 if the URA index is equal to 15

Note that accelerations represent only lunar and solar perturbation terms and satellite position can be computed based on equations in Section A.3.1.2 of GLONASS ICD version 5.0. See Appendix A14

8.3.5 RINEX navigation message files for BDS

The BDS Open Service broadcast navigation message; in general, is similar to the contents of the GPS navigation message

The header section and the first data record (epoch, satellite clock information) are equal to the GPS navigation file. The following seven records are similar to the GPS navigation.

The BDT week number is a continuous number. The broadcast 13-bit BDS System Time week has a roll-over after 8191. It starts at zero at : 1-Jan-2006, Hence BDT week = BDT week_BRD + (n*8192) (Where n: number of BDT roll-overs). See Appendix Table A13 for details.

8.4 RINEX observation files for GEO satellites

A separate satellite system identifier has been defined for the Satellite-Based Augmentation System (SBAS) payloads: **S**, to be used in the **RINEX VERSION / TYPE** header line and in the satellite identifier **snn**, **nn** being the GEO PRN number minus 100.

e.g.: PRN = 120 ⇒**snn** = **S20**

In mixed dual frequency GPS satellite / single frequency GEO payload observation files the fields for the second frequency observations of SBAS satellites remain blank, are set to zero values or (if last in the record) can be truncated.

The time system identifier of GEO satellites generating GPS signals defaults to GPS time. In the SBAS message definitions bit 3 of the health is currently marked as *reserved*. In case of bit 4 set to 1, it is recommended to set bits 0,1,2,3 to 1, as well.

User Range Accuracy (URA):

The same convention for converting the URA index to meters is used as with GPS. Set URA = 32767 meters if URA index = 15.

Issue Of Data Navigation (IODN)

The IODN is defined as the 8 first bits after the message type 9, called *IODN* in RTCA DO229, Annex A and Annex B and called *spare* in Annex C.

The **CORR TO SYSTEM TIME** header record has been replaced by the more general record **D-UTC. A0, A1, T, W, S, U** in Version 2.11.

9. MODIFICATIONS FOR VERSION 3.01 and 3.02

9.1 Phase Cycle Shifts

Carrier phases tracked on different signal channels or modulation bands of the same frequency may differ in phase by 1/4 (e.g., GPS: P/Y-code-derived L2 phase vs. L2C-based phase) or, in some exceptional cases, by other fractional parts of a cycle. Appendix Table19 specifies the reference signal and the phase shifts that are specified by the Interface Control Documentation (ICD) for each constellation.

All phase observations **must** be aligned in RINEX 3.01 and later files and the new **`SYS / PHASE SHIFT`** header is mandatory. See Appendix Table 2 for the messages definition. If the phase alignment is not known then the observation data **should not** be published in a RINEX 3.0x file. In order to facilitate data processing, phase observations stored in RINEX files **must** be consistent across all satellites of a satellite system and across each frequency band. Within a RINEX 3.0x file:

- Phase observations must be shifted by the respective fraction of a cycle, either directly by the receiver or by a correction program or the RINEX conversion program, prior to RINEX file generation, to align them to each other.
- Phase corrections must be reported in a new mandatory **`SYS / PHASE SHIFT`** header record to allow the reconstruction of the original values, if needed. The uncorrected reference signal group of observations are left blank in the **`SYS / PHASE SHIFT`** records. Appendix Table19 specifies the reference signal that should be used by each constellation and frequency band. Additionally, Appendix Table19 indicates the relationship between the phase observations for each frequency's signals.

Concerning the mandatory **`SYS / PHASE SHIFT header`** records:

- If the **`SYS / PHASE SHIFT`** record values are set to zero in the RINEX file, then either the raw data provided by the receiver or the data format (RTCM-Multiple Signal Messages format for example) have been already aligned the phase observations and the RINEX conversion program did not apply any phase corrections since they had already been applied. In this case Appendix Table19 can be used to determine the fractional cycles that had been added to each signal's phase observation to align the phase observations to the reference signal.
- If the file does not contain any observation pairs affected by phase shifts (i.e. only reference signals reported) the observation code field is defined and the rest of the **`SYS / PHASE SHIFT`** header record field of the respective satellite system(s) are left blank.
- If the reported phase correction of an observation type does not affect all satellites of the same system, the header record allows for the affected satellites to be indicated.

- If the applied phase corrections or the phase alignment is unknown the observation code field and the rest of the **SYS / PHASE SHIFT** header record field of the respective satellite system(s) are left blank. This use case is intended for exceptional situations where the data is intended for special projects and analysis.

Sign of the correction Δφ:

φRINEX	=	φ original	+	Δφ
φ original	:	Uncorrected or corrected, i.e. as issued by the GNSS receiver or in a standardized data stream such as RTCM-MSM		
Δφ	:	Phase correction to align the phase to other phases of the same frequency but different channel / modulation band		

Example (Definition see Appendix Table A2):

```
----|---1|0---|---2|0---|---3|0---|---4|0---|---5|0---|---6|0---|---7|0---|---8|
G L2S -0.25000  03 G15  G16 G17                               SYS / PHASE SHIFT
```

9.2 Galileo: BOC-Tracking of an MBOC-Modulated Signal

Galileo E1 will be modulated by the so-called MBOC modulation. Obviously it is possible for a receiver to track the signal also in a BOC mode, leading to different noise characteristics, though. In order to keep this non-standard tracking mode of a MBOC signal apart, bit 2 of the loss-of-lock indicator LLI (the antispoofing flag not used for Galileo) in the observation data records is used.

Non-standard BOC tracking of an MBOC-modulated signal: Increase the LLI by 4.

Example: Satellite E11, BOC tracking on L1C, LLI = 4:

```
----|---1|0---|---2|0---|---3|0---|---4|0---|---5|0---|---6|0---|---7|0---|---8|
G    5 C1C L1W L2W C1W S2W                                  SYS / # / OBS TYPES
R    2 C1C L1C                                              SYS / # / OBS TYPES
E    2 L1C L5I                                              SYS / # / OBS TYPES
S    2 C1C L1C                                              SYS / # / OBS TYPES
    18.000                                                  INTERVAL
                                                            END OF HEADER
> 2006 03 24 13 10 36.0000000  0  5      -0.123456789012
G06  23629347.915            .300 8         -.353 4  23629347.158          24.158
G09  20891534.648           -.120 9         -.358 6  20891545.292          38.123
G12  20607600.189           -.430 9          .394 5  20607600.848          35.234
E11          .32448          .178 7
S20  38137559.506      335849.135 9
```

9.3 BDS Satellite System Code

A satellite system code for BeiDou navigation satellite System (BDS) has been defined C, see Figure 1.

9.4 New Observation Codes for GPS L1C and BDS

New observation codes for GPS L1C and BDS observables have been defined: See Tables 2 and 7.

9.5 Header Records for GLONASS Slot and Frequency Numbers

In order to make available a cross-reference list between the GLONASS slot numbers used in the RINEX files to designate the GLONASS satellites and the allotted frequency numbers, an optional observation file header record is assigned. This allows processing of GLONASS files without having to get this information from GLONASS navigation message files or other sources.

Example (Definition See Appendix Table A2):

```
----|---1|0---|---2|0---|---3|0---|---4|0---|---5|0---|---6|0---|---7|0---|---8|
 18 R01  1 R02  2 R03  3 R04  4 R05  5 R06 -6 R07 -5 R08 -4 GLONASS SLOT / FRQ #
    R09 -3 R10 -2 R11 -1 R12  0 R13  1 R14  2 R15  3 R16  4 GLONASS SLOT / FRQ #
    R17  5 R18 -5                                           GLONASS SLOT / FRQ #
```

9.6 GNSS Navigation Message File: Leap Seconds Record

The optional **LEAP SECONDS** record was modified to also include TLS, WNLSF (adjusted to continuous week number) and DN.

9.7 Clarifications in the Galileo Navigation Message File:

Some clarifications in the Galileo ***BROADCAST ORBIT – 5*** and ***BROADCAST ORBIT – 6*** records were added (see Table A8).

9.8 RINEX Meteorological Data Files

The version number is adjusted to 3.01.

9.9 Added Quasi-Zenith Satellite System (QZSS) Version 3.02

The version number is adjusted to 3.02. Added QZSS : specifications, parameters and definitions to the documentation. Each QZSS satellite broadcasts signals using two PRN codes. The GPS compatible signals are broadcast using PRN codes in the range of 193-197. In a RINEX observation file the PRN code is: broadcast prn - 192, yielding: J01, J02 etc.. QZSS satellites also broadcasts a SBAS signal (QZSS-SAIF) using PRN codes in the range of 183-187. In a RINEX SBAS file the PRN code is: broadcast prn - 100, yielding: S83, S84 etc..

Added Appendix Table 19 to enable users to convert each signal's aligned phase observations back to raw satellite phase.

9.10 Added GLONASS Mandatory Code-Phase Alignment Header Record

Recent analysis has revealed that some GNSS receivers produce biased GLONASS observations. The code-phase bias results in the code and phase observations not being measured at the same time. To remedy this problem a mandatory GLONASS Code-Phase header bias record is required. Although this header message is mandatory it can contain zeros if the GLONASS data issued by the receiver is aligned. See the GLONASS CODE/PHASE BIAS (**GLONASS COD/PHS/BIS**) definition in Appendix Table A2. The GLONASS code-phase alignment message contains: L1C, L1P, L2C and L2P corrections. Phase data from GNSS receivers that issues biased data has to be corrected by the amount specified in the **GLONASS COD/PHS/BIS** record before it is written in RINEX format. To align the non-aligned L1C phase to the pseudo range observation the following correction is required: AlignedL1Cphase = ObservedL1Cphase + (GLONASSC1C_CodePhaseBias_M / Lambda) Where :

- AlignedL1Cphase in cycles (written to RINEX file)
- ObservedL1Cphase in cycles
- GLONASSC1C_CodePhaseBias_M is in metres;
- Lamba is the wavelength for the particular GLONASS frequency.

GLONASS L1P, L2C and L2P are handled in the same manner.

Example (Definition see Appendix Table A2 for details) :

```
----|---1|0---|---2|0---|---3|0---|---4|0---|---5|0---|---6|0---|---7|0---|---8|
 C1C  -10.000 C1P  -10.123 C2C  -10.432 C2P  -10.634        GLONASS COD/PHS/BIS#
```

9.11 Added BDS system (Replaces Compass)

Added BDS : naming convention, time system definition, header section description, and parameters through out the document. Updated: Sections: 8.1, 8.2, 8.3.5, 9.11 and Appendix Table A2, added ephemeris Table A13 and updated Table A19.

10. REFERENCES

Evans, A. (1989): “Summary of the Workshop on GPS Exchange Formats.” Proceedings of the Fifth International Geodetic Symposium on Satellite Systems, pp. 917ff, Las Cruces.

Gurtner, W., G. Mader, D. Arthur (1989): “A Common Exchange Format for GPS Data.” CSTG GPS Bulletin Vol.2 No.3, May/June 1989, National Geodetic Survey, Rockville.

Gurtner, W., G. Mader (1990a): “The RINEX Format: Current Status, Future Developments.” Proceedings of the Second International Symposium of Precise Positioning with the Global Positioning system, pp. 977ff, Ottawa.

Gurtner, W., G. Mader (1990b): “Receiver Independent Exchange Format Version 2.” CSTG GPS Bulletin Vol.3 No.3, Sept/Oct 1990, National Geodetic Survey, Rockville.

Gurtner, W. (1994): “RINEX: The Receiver-Independent Exchange Format.” GPS World, Volume 5, Number 7, July 1994.

Gurtner, W. (2002): “RINEX: The Receiver Independent Exchange Format Version 2.10”. ftp://igscb.jpl.nasa.gov/igscb/data/format/rinex210.txt

Gurtner, W., L. Estey (2002),: “RINEX Version 2.20 Modifications to Accommodate Low Earth Orbiter Data”. ftp://ftp.unibe.ch/aiub/rinex/rnx_leo.txt

Gurtner, W., L. Estey (2005): “RINEX: The Receiver Independent Exchange Format Version 2.11”. ftp://igscb.jpl.nasa.gov/igscb/data/format/rinex211.txt

Gurtner, W., L. Estey (2007): “RINEX: The Receiver Independent Exchange Format Version 3.00”. ftp://igscb.jpl.nasa.gov/igscb/data/format/rinex300.pdf

Hatanaka, Y (2008): “ A Compression Format and Tools for GNSS Observation Data”. Bulletin of the Geographical Survey Institute, Vol. 55, pp 21-30, Tsukuba, March 2008. http://www.gsi.go.jp/ENGLISH/Bulletin55.html

Ray, J., W. Gurtner (1999): “RINEX Extensions to Handle Clock Information”. ftp://igscb.jpl.nasa.gov/igscb/data/format/rinex_clock.txt.

Ray, J. (2005): “Final update for P1-C1 bias values & cc2noncc”. IGSMail 5260

Rothacher, M., R. Schmid (2005): “ANTEX: The Antenna Exchange Format Version 1.3”. ftp://igscb.jpl.nasa.gov/pub/station/general/antex13.txt.

Schaer, S., W. Gurtner, J. Feltens (1998): “IONEX: The Ionosphere Map Exchange Format Version 1“. ftp://igscb.jpl.nasa.gov/igscb/data/format/ionex1.pdf

Suard, N., W. Gurtner, L. Estey (2004): “Proposal for a new RINEX-type Exchange File for GEO SBAS Broadcast Data”. ftp://igscb.jpl.nasa.gov/igscb/data/format/geo_sbas.txt

Document RTCA DO 229, Appendix A

Document GAL OS SIS ICD/D.0: Galileo Interface Control Document, Revision 0, 23/05/2006, Chapter 9.

Quasi-Zenith Satellite System, Navigation Service, Interface Specification for QZSS (IS-QZSS), V1.4, Japan Aerospace Exploration Agency, February 28, 2012

BeiDou Navigation Satellite System, Signal In Space, Interface Control Document, Open Service Signal, B1I (Version1.0), China Satellite Navigation Office December 2012

APPENDIX: RINEX FORMAT DEFINITIONS AND EXAMPLES

A 1 RINEX File name description

Table A1

RINEX File name description

Field	Field Description	Example	Required	Comment/Example
<SITE/ STATION-MONUMENT/ RECEIVER/ COUNTRY/	XXXXMRCCC<br>Where:<br>XXXX - existing IGS station name<br>M – monument or marker number (0-9)<br>R – receiver number (0-9)<br>CCC – ISO Country code<br>(Total 9 characters)	ALGO00CAN	Yes	File name supports a maximum of 10 monuments at the same station and a maximum of 10 receivers per monument.<br><br>Country codes follow : ISO 3166-1 alpha-3
<DATA SOURCE>	Data Source<br>R – From Receiver data using vendor or other software<br>S – From data Stream (RTCM or other)<br>U – Unknown<br>(1 character)	R	Yes	This field is used to indicate how the data was collected either from the receiver at the station or from a data stream
<START TIME>	YYYYDDDHHMM<br>YYYY – Gregorian year 4 digits,<br>DDD – day of Year,<br>HHMM – hours and minutes of day<br><br>(11 characters)	2012150<br>1200	Yes	For GPS and Mixed files use : GPS Year, day of year, hour of day, minute of day (see text below for details)<br>Start time should be the nominal start time of the first observation.<br>GLONASS, Galileo etc use respective time system.
<FILE PERIOD>	DDU<br>DD – file period<br>U – units of file period.<br>File period is used to specify intended collection period of the file.<br>(3 characters)	15M	Yes	File Period<br>15M–15 Minutes<br>01H–1 Hour<br>01D–1 Day<br>01Y–1 Year<br>00U-Unspecified
<DATA FREQ>	DDU	05Z	Mandatory for	XXC – 100 Hertz<br>XXZ – HertZ,

Table A1
RINEX File name description

Field	Field Description	Example	Required	Comment/Example
	DD – data rate<br>U – units of data rate<br>(3 characters)		RINEX Obs. Data. NOT required for Navigatio n Files.	XXS – Seconds,<br>XXM – Minutes,<br>XXH – Hours,<br>XXD – Days<br>XXU – Unspecified
<DATA TYPE >	DD<br>DD – Data type<br><br>(2 characters)	MO	Yes	Two characters represent the data type:<br>GO - GPS Obs.,<br>RO - GLONASS Obs.,<br>EO - Galileo Obs.<br>JO - QZSS Obs.<br>CO - BDS Obs.<br>SO - SBAS Obs.<br>MO Mixed Obs.<br>GN - Nav. GPS,<br>RN- Glonass Nav.,<br>EN- Galileo Nav.,<br>JN- QZSS Nav.,<br>CN- BDS Nav.<br>SN- SBAS Nav.<br>MN- Nav. All GNSS Constellations)<br>MM-Meteorological Observation Etc
<FORMAT>	FFF<br>FFF – File format<br><br>(3 characters)	rnx	Yes	Three character indicating the data format :<br>RINEX - rnx,<br>Hatanaka Compressed RINEX – crx, ETC
<COMPRESSION>	(2-3 Characters)	gz	No	gz
Sub Total	34 or 35			Fields
Separators	(7 characters –Obs. File)<br>(6 characters –Eph. File)			_ under score between all fields and "." Between data type and file format and the compression method
Total	41-42(Obs. File)<br>37-38 (Eph. File)			Mandatory IGS RINEX obs. Characters

Filename Details and Examples:

<STATION/PROJECT NAME>: IGS users should follow XXXXMRCCC (9 char) site nd station naming convention described above.

GNSS industry users could use the 9 characters to indicate the project name and/or number.

<**DATA SOURCE**>: With real-time data streaming RINEX files for the same station can be created at many locations. If the RINEX file is derived from data collected at the receiver (official file) then the source is specified as R. On the other hand if the RINEX file is derived from a real-time data stream then the data source is marked as S to indicate Streamed data source. If the data source is unknown the source is marked as U.

<START TIME>: The start time is the file intended start time which should coincide with the first observation in the file. GPS file start time is specified in GPS Time. Mixed observation file start times are defined in GPS Time. Files containing only: GLONASS, Galileo, QZSS, BDS or SBAS observations are all based on their respective time system.

<FILE PERIOD>: Is used to specify the intended collection period of the file.

GNSS observation file name - file period examples:

ALGO00CAN_R_20121601000_15M_01S_GO.rnx.gz //15 min, GPS Obs. 1 sec.
ALGO00CAN_R_20121601000_01H_05Z_MO.rnx.gz //1 hour, Obs Mixed and 5Hz
ALGO00CAN_R_20121601000_01D_30S_GO.rnx.gz //1 day, Obs GPS and 30 sec
ALGO00CAN_R_20121601000_01D_30S_MO.rnx.gz //1 day, Obs. Mixed, 30 sec

GNSS navigation file name - file period examples:

ALGO00CAN_R_20121600000_15M_GN.rnx.gz // 15 minute GPS only
ALGO00CAN_R_20121600000_01H_GN.rnx.gz // 1 hour GPS only
ALGO00CAN_R_20121600000_01D_MN.rnx.gz // 1 day mixed

<DATA FREQ>: Used to distinguish between observation files that cover the same period but contain data at a different sampling rate. GNSS data file - observation frequency examples:

ALGO00CAN_R_20121601000_01D_01C_GO.rnx.gz //100 Hz data rate
ALGO00CAN_R_20121601000_01D_05Z_RO.rnx.gz //5 Hz data rate
ALGO00CAN_R_20121601000_01D_01S_EO.rnx.gz //1 second data rate
ALGO00CAN_R_20121601000_01D_05M_JO.rnx.gz //5 minute data rate
ALGO00CAN_R_20121601000_01D_01H_CO.rnx.gz //1 hour data rate

ALGO00CAN_R_20121601000_01D_01D_SO.rnx.gz //1 day data rate
ALGO00CAN_R_20121601000_01D_00U_MO.rnx.gz //Unspecified

Note : Data frequency field not required for RINEX Navigation files.

< DATA TYPE/ FORMAT/>: The data type describes the content of the file. The first character indicates constellation and the second indicates whether the files contains observations or navigation data. The next three characters indicate the data file format. GNSS observation filename - format/data type examples:

ALGO00CAN_R_20121601000_15M_01S_GO.rnx.gz //RINEX obs. GPS
ALGO00CAN_R_20121601000_15M_01S_RO.rnx.gz //RINEX obs. GLONASS
ALGO00CAN_R_20121601000_15M_01S_EO.rnx.gz //RINEX obs. Galileo
ALGO00CAN_R_20121601000_15M_01S_JO.rnx.gz //RINEX obs. QZSS
ALGO00CAN_R_20121601000_15M_01S_CO.rnx.gz //RINEX obs. BDS
ALGO00CAN_R_20121601000_15M_01S_SO.rnx.gz //RINEX obs. SBAS
ALGO00CAN_R_20121601000_15M_01S_MO.rnx.gz //RINEX obs. mixed

GNSS navigation filename examples:

ALGO00CAN_R_20121600000_01H_GN.rnx.gz //RINEX nav. GPS
ALGO00CAN_R_20121600000_01H_RN.rnx.gz //RINEX nav. GLONASS
ALGO00CAN_R_20121600000_01H_EN.rnx.gz //RINEX nav. Galileo
ALGO00CAN_R_20121600000_01H_JN.rnx.gz //RINEX nav. QZSS
ALGO00CAN_R_20121600000_01H_CN.rnx.gz //RINEX nav. BDS
ALGO00CAN_R_20121600000_01H_SN.rnx.gz //RINEX nav. SBAS
ALGO00CAN_R_20121600000_01H_MN.rnx.gz //RINEX nav. mixed

<COMPRESSION>:

Valid compression methods include: UNIX Compress ".Z", gzip - ".gz", bzip2 - ".bz2" and ".zip".

A 2 GNSS Observation Data File -Header Section Description

TABLE A2<br>GNSS OBSERVATION DATA FILE - HEADER SECTION DESCRIPTION		
**HEADER LABEL**<br>(Columns 61-80)	**DESCRIPTION**	**FORMAT**
**RINEX VERSION / TYPE**	Format version : **3.02**<br>File type: **O** for Observation Data<br>- Satellite System:<br>- **G**: GPS<br>- **R**: GLONASS<br>- **E**: Galileo<br>- **J**: QZSS<br>- **C:** BDS<br>- **S**: SBAS payload<br>- **M**: Mixed	F9.2, 11X,<br>A1,19X,<br>A1,19X
**PGM / RUN BY / DATE**	- Name of program creating current file<br>- Name of agency creating current file<br>- Date and time of file creation<br>Format: yyyymmdd hhmmss zone<br>zone: 3-4 char. code for time zone.<br>'**UTC** ' recommended!<br>'**LCL** ' if local time with unknown local time system code	A20,<br>A20,<br>A20
*** COMMENT**	Comment line(s)	A60
**MARKER NAME**	Name of antenna marker	A60
*** MARKER NUMBER**	Number of antenna marker	A20
**MARKER TYPE**	Type of the marker:<br>**GEODETIC** : Earth-fixed, high- precision monument<br>**NON_GEODETIC** : Earth-fixed, low- precision monument<br>**NON_PHYSICAL** : Generated from network processing<br>**SPACEBORNE** : Orbiting space vehicle<br>**GROUND_CRAFT** : Mobile terrestrial vehicle<br>**WATER_CRAFT** : Mobile water craft<br>**AIRBORNE:** Aircraft, balloon, etc.<br>**FIXED_BUOY** : "Fixed" on water surface<br>**FLOATING_BUOY**: Floating on water surface<br>**FLOATING_ICE** : Floating ice sheet, etc.<br>**GLACIER** : "Fixed" on a glacier<br>**BALLISTIC** : Rockets, shells, etc	A20,40X

TABLE A2<br>GNSS OBSERVATION DATA FILE - HEADER SECTION DESCRIPTION		
	**ANIMAL** : Animal carrying a receiver<br>**HUMAN** : Human being<br>Record required except for **GEODETIC** and **NON_GEODETIC** marker types. Users may define other project-dependent keywords.	
**OBSERVER / AGENCY**	Name of observer / agency	A20,A40
**REC # / TYPE / VERS**	Receiver number, type, and version (Version: e.g. Internal Software Version)	3A20
**ANT # / TYPE**	Antenna number and type	2A20
**APPROX POSITION XYZ**	Geocentric approximate marker position (Units: Meters, System: ITRS recommended) Optional for moving platforms	3F14.4
**ANTENNA: DELTA H/E/N**	- Antenna height: Height of the antenna reference point (ARP) above the marker<br>- Horizontal eccentricity of ARP relative to the marker (east/north)<br>All units in meters	F14.4,<br>2F14.4
*** ANTENNA: DELTA X/Y/Z**	Position of antenna reference point for antenna on vehicle (m): XYZ vector in body-fixed coord. system	3F14.4
***ANTENNA:PHASE CENTER**	Average phase center position w/r to antenna reference point (m)<br>- Satellite system (**G**/**R**/**E**/**J**/**C**/**S**)<br>- - Observation code<br>- - North/East/Up (fixed station) or<br>- X/Y/Z in body-fixed system (vehicle)	 <br>A1,<br>1X,A3,<br>F9.4,<br>2F14.4
*** ANTENNA: B.SIGHT XYZ**	Direction of the “vertical” antenna axis towards the GNSS satellites.<br>Antenna on vehicle: Unit vector in body-fixed coord. System<br>Tilted antenna on fixed station: Unit vector in N/E/Up left-handed system	3F14.4
*** ANTENNA: ZERODIR AZI**	Azimuth of the zero-direction of a fixed antenna (degrees, from north)	F14.4
*** ANTENNA: ZERODIR XYZ**	Zero-direction of antenna<br>Antenna on vehicle: Unit vector in body-fixed coord. system<br>Tilted antenna on fixed station: Unit vector in N/E/Up left-handed system	3F14.4
*** CENTER OF MASS: XYZ**	Current center of mass (X,Y,Z, meters) of vehicle in body-fixed coordinate system. Same system as used for attitude.	3F14.4

TABLE A2<br>GNSS OBSERVATION DATA FILE - HEADER SECTION DESCRIPTION		
**SYS / # / OBS TYPES**	- Satellite system code (**G/R/E/J/C/S/M**)<br>- Number of different observation types for the specified satellite system<br>- Observation descriptors:<br>o Type<br>o Band<br>o Attribute<br>Use continuation line(s) for more than 13 observation descriptors.<br>In mixed files: Repeat for each satellite system.<br>These records should precede any **SYS / SCALE FACTOR** records (see below).<br>The following observation descriptors are defined in RINEX Version 3.xx:<br>Type:<br>**C** = Code / Pseudorange<br>**L** = Phase<br>**D** = Doppler<br>**S** = Raw signal strength(carrier to noise ratio)<br>**I** = Ionosphere phase delay<br>**X** = Receiver channel numbers<br>Band:<br>**1** = L1 (GPS, QZSS, SBAS)<br>G1 (GLO)<br>E2-L1-E1 (GAL)<br>B1 (BDS)<br>**2** = L2 (GPS, QZSS)<br>G2 (GLO)<br>**5** = L5 (GPS, QZSS, SBAS)<br>E5a (GAL)<br>**6** = E6 (GAL)<br>LEX (QZSS)<br>B3 (BDS)<br>**7** = E5b (GAL)<br>B2 (BDS)<br>**8** = E5a+b (GAL)<br>**0** for type **X** (all)	A1,<br>2X,I3,<br><br>13(1X,A3)<br><br><br><br>6X,<br>13(1X,A3)
**SYS / # / OBS TYPES**<br>(Continued)	Attribute:<br>**P** = P code-based (GPS,GLO)<br>**C** = C code-based (SBAS,GPS,GLO, QZSS)<br>**D** = semi-codeless (GPS)<br>**Y** = Y code-based (GPS)	

TABLE A2<br>GNSS OBSERVATION DATA FILE - HEADER SECTION DESCRIPTION		
	**M** = M code-based (GPS)<br>**N** = codeless (GPS)<br>**A** = A channel (GAL)<br>**B** = B channel (GAL)<br>**C** = C channel (GAL)<br>**I** = I channel (GPS,GAL, QZSS, BDS)<br>**Q** = Q channel (GPS,GAL, QZSS, BDS)<br>**S** = M channel (L2C GPS, QZSS)<br>**L** = L channel (L2C GPS, QZSS)<br>**S** = D channel (GPS, QZSS)<br>**L** = P channel (GPS, QZSS)<br><br>**X** = B+C channels (GAL)<br>I+Q channels (GPS,GAL, QZSS, BDS)<br>M+L channels (GPS, QZSS)<br>D+P channels (GPS, QZSS)<br>**W** = based on Z-tracking (GPS) (see text)<br>**Z** = A+B+C channels (GAL)<br>***blank*** : for types **I** and **X** (all) or unknown tracking mode<br>All characters in uppercase only!<br><br>Units :<br>Phase : full cycles<br>Pseudorange : meters<br>Doppler : Hz<br>SNR etc : receiver-dependent<br>Ionosphere : full cycles<br>Channel # : See text<br><br>Sign definition: See text.<br><br>The sequence of the observations in the observation records has to correspond to the sequence of the types in this record of the respective satellite system.<br><br>The attribute can be left blank if not known. See text!	
*** SIGNAL STRENGTH UNIT**	Unit of the carrier to noise ratio observables **Snn** (if present) **DBHZ** : S/N given in dbHz<br>...	A20,40X

TABLE A2<br>GNSS OBSERVATION DATA FILE - HEADER SECTION DESCRIPTION		
*** INTERVAL**	Observation interval in seconds	F10.3
**TIME OF FIRST OBS**	- Time of first observation record (4-digit-year, month, day, hour, min, sec)<br>Time system:<br>- **GPS** (=GPS time system)<br>- **GLO** (=UTC time system)<br>- **GAL** (=Galileo System Time)<br>- **QZS** (= QZSS time system)<br>- **BDT** (=BDS Time system)<br>Compulsory in mixed GNSS files<br>Defaults:<br>**GPS** for pure GPS files<br>**GLO** for pure GLONASS files<br>**GAL** for pure Galileo files<br>**QZS** for pure QZSS files<br>**BDT** for pure BDS files	5I6,F13.7,<br><br><br>5X,A3
*** TIME OF LAST OBS**	- Time of last observation record (4-digit-year, month,day,hour,min,sec)<br>- Time system: Same value as in **TIME OF FIRST OBS** record	5I6,F13.7,<br><br>5X,A3
*** RCV CLOCK OFFS APPL**	Epoch, code, and phase are corrected by applying the realtime-derived receiver clock offset: 1=yes, 0=no; default: 0=no Record required if clock offsets are reported in the **EPOCH/SAT** records	I6
*** SYS / DCBS APPLIED**	- Satellite system (**G**/**R**/**E**/**J**/**C**/**S**)<br>- Program name used to apply differential code bias corrections<br>- Source of corrections (URL)<br>Repeat for each satellite system.<br>No corrections applied: Blank fields or record not present.	A1,<br>1X,A17,<br><br>1X,A40
*** SYS / PCVS APPLIED**	- Satellite system (**G**/**R**/**E**/**J**/**C**/**S**)<br>- Program name used to apply phase center variation corrections<br>- Source of corrections (URL)<br>Repeat for each satellite system.<br>No corrections applied: Blank fields or record not present.	A1,<br>1X,A17,<br><br>1X,A40
*** SYS / SCALE FACTOR**	- Satellite system (**G**/**R**/**E**/**J**/**C**/**S**)<br>- Factor to divide stored observations with before use (1,10,100,1000)<br>- Number of observation types involved. 0 or	A1,<br>1X,I4,<br><br>2X,I2,

TABLE A2<br>GNSS OBSERVATION DATA FILE - HEADER SECTION DESCRIPTION		
	blank: All observation types<br>- List of observation types<br><br>Use continuation line(s) for more than 12 observation types.<br><br>Repeat record if different factors are applied to different observation types.<br><br>A value of 1 is assumed if record is missing.	 12(1X,A3)<br><br>10X,<br>12(1X,A3)
**SYS / PHASE SHIFT**	Phase shift correction used to generate phases consistent w/r to cycle shifts<br>- Satellite system (**G**/**R**/**E**/**J**/**C**/**S**)<br>- Carrier phase observation code<br>- Type<br>- Band<br>- Attribute<br>- Correction applied (cycles)<br>- Number of satellites involved 0 or blank: All satellites of system<br>- List of satellites<br>Use continuation line(s) for more than 10 satellites.<br>Repeat the record for all affected codes.<br><br>Leave observation code and rest of the field blank if applied corrections for the respective satellite system are unknown.<br>phase(RINEX) = phase(orig) + correction. See chapter 9.1!	 <br>A1,1X,<br>A3,1X,<br><br><br><br>F8.5<br>2X,I2.2,<br><br>10(1X,A3)<br>18X,<br>10(1X,A3)
**GLONASS SLOT / FRQ #**	GLONASS slot and frequency numbers<br>- Number of satellites in list<br>List of :<br>- Satellite numbers (system code, slot)<br>- Frequency numbers (-7...+6)<br>Use continuation lines for more than 8 Satellites	 I3,1X,<br><br>8(A1,I2.2,<br>1X,I2,1X)<br>4X,8(A1,<br>I2.2,1X,I2,1<br>X)
**GLONASS COD/PHS/BIS**	GLONASS Phase bias correction used to align code and phase observations.<br>- GLONASS signal identifier : C1C and Code Phase bias correction (metres)<br>- GLONASS signal identifier : C1P and Code Phase bias correction (metres)<br>- GLONASS signal identifier : C2C and Code	4(X1,A3,X1,<br>F8.3)

TABLE A2<br>GNSS OBSERVATION DATA FILE - HEADER SECTION DESCRIPTION		
	Phase bias correction (metres)<br>- GLONASS signal identifier : C2P and Code Phase bias correction (metres)	
*** LEAP SECONDS**	- Number of leap seconds since 6-Jan-1980 as transmitted by the GPS almanac _t_LS<br>- Future or past leap seconds _t_LSF<br>- Respective week number WN_LSF (continuous number)<br>- Respective day number DN<br>(see ICD-GPS-200C 20.3.3.5.2.4)<br>Zero or blank if not known	I6,<br><br>I6,<br>I6,<br><br>I6
*** # OF SATELLITES**	Number of satellites, for which observations are stored in the file	I6
*** PRN / # OF OBS**	Satellite numbers, number of observations for each observation type indicated in the **SYS / # / OBS TYPES** record.<br><br>If more than 9 observation types:<br>Use continuation line(s)<br>In order to avoid format overflows, 99999 indicates >= 99999 observations in the RINEX file.<br><br>This record is (these records are) repeated for each satellite present in the data file.	3X,<br>A1,I2.2,<br>9I6<br><br>6X,9I6
**END OF HEADER**	Last record in the header section.	60X

Records marked with * are optional

A 3 GNSS Observation Data File -Data Record Description

TABLE A3<br>GNSS OBSERVATION DATA FILE – DATA RECORD DESCRIPTION	
**DESCRIPTION**	**FORMAT**
EPOCH record	
- Record identifier : >	A1,
- Epoch	
- year (4 digits):	1X,I4,
- month, day, hour, min (two digits)	4(1X,I2.2),
- sec	F11.7,
- Epoch flag<br>0: OK<br>1: power failure between previous and current epoch<br>>1: Special event	2X,I1,
- Number of satellites observed in current epoch	I3,
- (reserved)	6X,
- Receiver clock offset (seconds, optional)	F15.12,
Epoch flag = 0 or 1: ***OBSERVATION*** records follow	
- Satellite number	A1,I2.2,
- Observation - repeat within record for each observation	m(F14.3,
- LLI - type (same sequence as given in the respective **SYS / # / OBS TYPES** record)	I1,
- Signal strength	I1)
This record is repeated for each satellite having been observed in the current epoch. The record length is given by the number of observation types for this satellite.<br>Observations: For definition see text.<br>Missing observations are written as 0.0 or blanks.Phase values overflowing the fixed format F14.3 have to be clipped into the valid interval (e.g add or subtract 10**9), set bit 0 of LLI indicator.<br>Loss of lock indicator (LLI).<br>0 or blank: OK or not known<br><br>Bit 0 set : Lost lock between previous and current observation: Cycle slip possible. For phase observations only.<br><br>Bit 1 set : Half-cycle ambiguity/slip possible. Software not capable of handling half cycles should skip this observation. Valid for the current epoch only.<br><br>Bit 2 set : Galileo BOC-tracking of an MBOC-modulated signal (may suffer from increased noise).	

TABLE A3<br>GNSS OBSERVATION DATA FILE – DATA RECORD DESCRIPTION	
Signal strength projected into interval 1-9:<br>1: minimum possible signal strength<br>5: average S/N ratio<br>9: maximum possible signal strength<br>0 or blank: not known, don't care<br>Standardization for S/N values given in dbHz: See text.	
- Epoch flag 2-5: ***EVENT***: *Special records* may follow<br>- Epoch flag<br>- 2: start moving antenna<br>- 3: new site occupation (end of kinematic data) (at least MARKER NAME record follows)<br>- 4: header information follows<br>- 5: external event (epoch is significant, same time frame as observation time tags)<br>- "Number of satellites" contains number of special records to follow. 0 if no special records follow.<br>- Maximum number of records: 999<br><br>For events without significant epoch the epoch fields in the EPOCH RECORD can be left blank	[2X,I1]<br><br><br><br><br><br>[I3]
Epoch flag = 6: ***EVENT***: *Cycle slip records* follow<br>- Epoch flag<br>- 6: cycle slip records follow to optionally report detected and repaired cycle slips (same format as ***OBSERVATIONS*** records;<br>o slip instead of observation;<br>o LLI and signal strength blank or zero)	[2X,I1]

A 4 GNSS Observation Data File - Example

```
+------------------------------------------------------------------------------+
|                                TABLE A4                                      |
|                   GNSS OBSERVATION DATA FILE - EXAMPLE                       |
+------------------------------------------------------------------------------+

----|---1|0---|---2|0---|---3|0---|---4|0---|---5|0---|---6|0---|---7|0---|---8|0-

     3.02           OBSERVATION DATA    M                   RINEX VERSION / TYPE
G = GPS  R = GLONASS  E = GALILEO  S = GEO  M = MIXED       COMMENT
XXRINEXO V9.9       AIUB                20060324 144333 UTC PGM / RUN BY / DATE
EXAMPLE OF A MIXED RINEX FILE VERSION 3.02                  COMMENT
The file contains L1 pseudorange and phase data of the      COMMENT
geostationary AOR-E satellite (PRN 120 = S20)               COMMENT
A 9080                                                      MARKER NAME
9080.1.34                                                   MARKER NUMBER
BILL SMITH          ABC INSTITUTE                           OBSERVER / AGENCY
X1234A123           GEODETIC            1.3.1               REC # / TYPE / VERS
G1234               ROVER                                   ANT # / TYPE
  4375274.       587466.      4589095.                      APPROX POSITION XYZ
         .9030          .0000          .0000                ANTENNA: DELTA H/E/N
     0                                                      RCV CLOCK OFFS APPL
G    5 C1C L1W L2W C1W S2W                                  SYS / # / OBS TYPES
R    2 C1C L1C                                              SYS / # / OBS TYPES
E    2 L1B L5I                                              SYS / # / OBS TYPES
S    2 C1C L1C                                              SYS / # / OBS TYPES
    18.000                                                  INTERVAL
G APPL_DCB          xyz.uvw.abc//pub/dcb_gps.dat            SYS / DCBS APPLIED
DBHZ                                                        SIGNAL STRENGTH UNIT
  2006    03    24    13    10   36.0000000     GPS         TIME OF FIRST OBS
 18 R01  1 R02  2 R03  3 R04  4 R05  5 R06 -6 R07 -5 R08 -4 GLONASS SLOT / FRQ #
    R09 -3 R10 -2 R11 -1 R12  0 R13  1 R14  2 R15  3 R16  4 GLONASS SLOT / FRQ #
    R17  5 R18 -5                                           GLONASS SLOT / FRQ #
 C1C  -10.000 C1P  -10.123 C2C  -10.432 C2P  -10.634        GLONASS COD/PHS/BIS
                                                            END OF HEADER
> 2006 03 24 13 10 36.0000000  0  5      -0.123456789012
G06  23629347.915            .300 8         -.353 4  23629347.158          24.158
G09  20891534.648           -.120 9         -.358 6  20891545.292          38.123
G12  20607600.189           -.430 9          .394 5  20607600.848          35.234
E11          .324 8          .178 7
S20  38137559.506      335849.135 9
> 2006 03 24 13 10 54.0000000  0  7      -0.123456789210
G06  23619095.450      -53875.632 8    -41981.375 4  23619095.008          25.234
G09  20886075.667      -28688.027 9    -22354.535 7  20886076.101          42.231
G12  20611072.689       18247.789 9     14219.770 6  20611072.410          36.765
R21  21345678.576       12345.567 5
R22  22123456.789       23456.789 5
E11     65432.123 5     48861.586 7
S20  38137559.506      335849.135 9
> 2006 03 24 13 11 12.0000000  2  2
      *** FROM NOW ON KINEMATIC DATA! ***                   COMMENT
      TWO COMMENT LINES FOLLOW DIRECTLY THE EVENT RECORD    COMMENT
> 2006  3 24 13 11 12.0000000  0  4      -0.123456789876
G06  21110991.756       16119.980 7     12560.510 4  21110991.441          25.543
G09  23588424.398     -215050.557 6   -167571.734 6  23588424.570          41.824
G12  20869878.790     -113803.187 8    -88677.926 6  20869878.938          36.961
G16  20621643.727       73797.462 7     57505.177 2  20621644.276          15.368
>                               3  4
A 9081                                                      MARKER NAME
```

```
9081.1.34                                                   MARKER NUMBER
         .9050         .0000         .0000                  ANTENNA: DELTA H/E/N
          --> THIS IS THE START OF A NEW SITE <--           COMMENT
> 2006 03 24 13 12  6.0000000  0  4      -0.123456987654
G06  21112589.384       24515.877 6     19102.763 4  21112589.187          25.478
G09  23578228.338     -268624.234 7   -209317.284 6  23578228.398          41.725
G12  20625218.088       92581.207 7     72141.846 5  20625218.795          35.143
G16  20864539.693     -141858.836 8   -110539.435 2  20864539.943          16.345
> 2006 03 24 13 13  1.2345678  5  0
>                              4  2
      AN EVENT FLAG 5 WITH A SIGNIFICANT EPOCH              COMMENT
  AND AN EVENT FLAG 4 TO ESCAPE FOR THE TWO COMMENT LINES   COMMENT
> 2006 03 24 13 14 12.0000000  0  4      -0.123456012345
G06  21124965.133           0.30213        -0.62614  21124965.275          27.528
G09  23507272.372     -212616.150 7   -165674.789 7  23507272.421          42.124
G12  20828010.354     -333820.093 6   -260119.395 6  20828010.129          37.002
G16  20650944.902      227775.130 7    177487.651 3  20650944.363          18.040
>                              4  1
           *** LOST LOCK ON G 06                            COMMENT
 .
 .
 .
>                              4  1
END OF FILE                                                 COMMENT
----|---1|0---|---2|0---|---3|0---|---4|0---|---5|0---|---6|0---|---7|0---|---8|0-
```

A 5 GNSS Navigation Message File – Header Section Description

TABLE A5<br>GNSS NAVIGATION MESSAGE FILE - HEADER SECTION DESCRIPTION		
**HEADER LABEL**<br>(Columns 61-80)	**DESCRIPTION**	**FORMAT**
**RINEX VERSION / TYPE**	- Format version : **3.02**<br>File type ('**N**' for navigation data)<br>Satellite System:<br>**G**: GPS<br>**R**: GLONASS<br>**E**: Galileo<br>**J**: QZSS<br>**C**: BDS<br>**S**: SBAS Payload<br>**M**: Mixed	F9.2,11X,<br>A1,19X,<br><br>A1,19X
**PGM / RUN BY / DATE**	Name of program creating current file<br>Name of agency creating current file<br>Date and time of file creation<br>Format: yyyymmdd hhmmss zone zone: 3-4 char. code for time zone.<br>'**UTC** ' recommended!<br>'**LCL** ' if local time with unknown local time system code	A20,<br>A20,<br><br>A20
*** COMMENT**	Comment line(s)	A60
*** IONOSPHERIC CORR**	Ionospheric correction parameters<br>Correction type<br>**GAL** = Galileo ai0 – ai2<br>**GPSA** = GPS alpha0 - alpha3<br>**GPSB** = GPS beta0 - beta3<br>**QZSA** = QZS alpha0 - alpha3<br>**QZSB** = QZS beta0 - beta3<br>**BDSA** = BDS alpha0 - alpha3<br>**BDSB** = BDS beta0 - beta3<br>Parameters<br>GPS: alpha0-alpha3 or beta0-beta3<br>GAL: ai0, ai1, ai2, zero<br>QZS: alpha0-alpha3 or beta0-beta3<br>BDS: alpha0-alpha3 or beta0-beta3	A4,1X,<br><br><br><br><br>4D12.4
*** TIME SYSTEM CORR**	Corrections to transform the system time to UTC or other time systems<br>Correction type<br>**GAUT** = GAL to UTC a0, a1<br>**GPUT** = GPS to UTC a0, a1	A4,1X,

TABLE A5<br>GNSS NAVIGATION MESSAGE FILE - HEADER SECTION DESCRIPTION		
**HEADER LABEL**<br>(Columns 61-80)	**DESCRIPTION**	**FORMAT**
	**SBUT** = SBAS to UTC a0, a1<br>**GLUT** = GLO to UTC a0=TauC, a1=zero<br>**GPGA** = GPS to GAL a0=A0G, a1=A1G<br>**GLGP** = GLO to GPS a0=TauGPS, a1=zero<br>**QZGP** = QZS to GPS a0, a1<br>**QZUT** = QZS to UTC a0, a1<br>**BDUT** =BDS to UTC a0=A_{0UTC}, a1=A_{1UTC}<br><br><br>a0,a1 Coefficients of 1-deg polynomial (a0 sec, a1 sec/sec) CORR(s) = a0 + a1*DELTAT<br>T Reference time for polynomial (Seconds into GPS/GAL week)<br>W Reference week number (GPS/GAL week, continuous number) T and W zero for GLONASS.<br>S **EGNOS**, **WAAS**, or **MSAS** ... (left-justified) Derived from MT17 service provider. If not known: Use **Snn** with:<br>nn = PRN-100 of satellite broadcasting the MT12<br>U UTC Identifier (0 if unknown) 1=UTC(NIST), 2=UTC(USNO), 3=UTC(SU), 4=UTC(BIPM), 5=UTC(Europe Lab), 6=UTC(CRL), 7=UTC(NTSC) (BDS), >7 = not assigned yet S and U for SBAS only.	D17.10,<br>D16.9,<br><br>I7,<br><br>I5,<br><br>1X,A5,1X<br><br><br>I2,1X
*** LEAP SECONDS**	Number of leap seconds since 6-Jan-1980 as transmitted by the GPS almanac or since 1-Jan-2006 as transmitted by BDS almanac _t_LS,<br>Future or past leap seconds _t_LSF<br>Respective week number WN_LSF (continuous number)<br>Respective day number DN (see ICD-GPS-200C 20.3.3.5.2.4)<br>Zero or blank if not known	I6,<br><br><br>I6,<br>I6,<br><br>I6
**END OF HEADER**	Last record in the header section.	60X

Records marked with * are optional

A 6 GNSS Navigation Message File – GPS Data Record Description

TABLE A6<br>GNSS NAVIGATION MESSAGE FILE – GPS/QZSS DATA RECORD DESCRIPTION		
**OBS. RECORD**	**DESCRIPTION**	**FORMAT**
SV / EPOCH / SV CLK	- Satellite system (**G/J**), sat number (PRN, (QZSS: PRN-192))<br>- Epoch: Toc - Time of Clock (GPS) year (4 digits)<br>- month, day, hour, minute, second<br>- SV clock bias (seconds)<br>- SV clock drift (sec/sec)<br>- SV clock drift rate (sec/sec2)	A1,I2.2,<br><br>1X,I4,<br>5(1X,I2.2),<br>3D19.12<br><br>*)
**BROADCAST ORBIT - 1**	- IODE Issue of Data, Ephemeris<br>- Crs (meters)<br>- Delta n (radians/sec)<br>- M0 (radians)	4X,4D19.12<br><br>***)
**BROADCAST ORBIT - 2**	- Cuc (radians)<br>- e Eccentricity<br>- Cus (radians)<br>- sqrt(A) (sqrt(m))	4X,4D19.12
**BROADCAST ORBIT - 3**	- Toe Time of Ephemeris (sec of GPS week)<br>- Cic (radians)<br>- OMEGA0 (radians)<br>- Cis (radians)	4X,4D19.12
**BROADCAST ORBIT - 4**	- i0 (radians)<br>- Crc (meters)<br>- omega (radians)<br>- OMEGA DOT (radians/sec)	4X,4D19.12
**BROADCAST ORBIT - 5**	- IDOT (radians/sec)<br>- Codes on L2 channel<br>- GPS Week # (to go with TOE) Continuous number, not mod(1024)!<br>- L2 P data flag	4X,4D19.12
**BROADCAST ORBIT - 6**	- SV accuracy (meters)<br>- SV health (bits 17-22 w 3 sf 1)<br>- TGD (seconds)<br>- IODC Issue of Data, Clock	4X,4D19.12
**BROADCAST ORBIT - 7**	- Transmission time of message **)<br>(sec of GPS week, derived e.g.from Z-count in Hand Over Word (HOW))<br>- Fit interval (hours) (see ICD-GPS-200, 20.3.4.4) 0 = 4 hours, 1 = 6 hours<br>- Spare<br>- Spare	4X,4D19.12

*) In order to account for the various compilers, E,e,D, and d are allowed letters between the

fraction and exponent of all floating point numbers in the navigation message files. Zero-padded two-digit exponents are required, however.

) Adjust the *Transmission time of message* by + or -604800 to refer to the reported week in ***BROADCAST ORBIT 5*, if necessary. Set value to 0.9999E9 if not known.

A 7 GPS Navigation Message File – Example

```
+------------------------------------------------------------------------------+
|                                  TABLE A7                                    |
|                    GPS NAVIGATION MESSAGE FILE - EXAMPLE                     |
+------------------------------------------------------------------------------+

----|---1|0---|---2|0---|---3|0---|---4|0---|---5|0---|---6|0---|---7|0---|---8|

     3.02           N: GNSS NAV DATA    G: GPS              RINEX VERSION / TYPE
XXRINEXN V3         AIUB                19990903 152236 UTC PGM / RUN BY / DATE
EXAMPLE OF VERSION 3.02 FORMAT                              COMMENT
GPSA    .1676D-07   .2235D-07   .1192D-06   .1192D-06       IONOSPHERIC CORR
GPSB    .1208D+06   .1310D+06  -.1310D+06  -.1966D+06       IONOSPHERIC CORR
GPUT   .1331791282D-06  .107469589D-12 552960 1025          TIME SYSTEM CORR
    13                                                      LEAP SECONDS
                                                            END OF HEADER
G06 1999 09 02 17 51 44 -.839701388031D-03 -.165982783074D-10  .000000000000D+00
      .910000000000D+02  .934062500000D+02  .116040547840D-08  .162092304801D+00
      .484101474285D-05  .626740418375D-02  .652112066746D-05  .515365489006D+04
      .409904000000D+06 -.242143869400D-07  .329237003460D+00 -.596046447754D-07
      .111541663136D+01  .326593750000D+03  .206958726335D+01 -.638312302555D-08
      .307155651409D-09  .000000000000D+00  .102500000000D+04  .000000000000D+00
      .000000000000D+00  .000000000000D+00  .000000000000D+00  .910000000000D+02
      .406800000000D+06  .000000000000D+00
G13 1999 09 02 19 00 00  .490025617182D-03  .204636307899D-11  .000000000000D+00
      .133000000000D+03 -.963125000000D+02  .146970407622D-08  .292961152146D+01
     -.498816370964D-05  .200239347760D-02  .928156077862D-05  .515328476143D+04
      .414000000000D+06 -.279396772385D-07  .243031939942D+01 -.558793544769D-07
      .110192796930D+01  .271187500000D+03 -.232757915425D+01 -.619632953057D-08
     -.785747015231D-11  .000000000000D+00  .102500000000D+04  .000000000000D+00
      .000000000000D+00  .000000000000D+00  .000000000000D+00  .389000000000D+03
      .410400000000D+06  .000000000000D+00

----|---1|0---|---2|0---|---3|0---|---4|0---|---5|0---|---6|0---|---7|0---|---8|
```

A 8 GNSS Navigation Message File – GALILEO Data Record Description

TABLE A8<br>GNSS NAVIGATION MESSAGE FILE - GALILEO DATA RECORD DESCRIPTION		
**OBS. RECORD**	**DESCRIPTION**	**FORMAT**
**SV / EPOCH / SV CLK**	- Satellite system (**E**), satellite number<br>- Epoch: Toc - Time of Clock GALyear (4 digits)<br>- month, day, hour, minute, second<br>- SV clock bias (seconds) af0<br>- SV clock drift (sec/sec) af1<br>- SV clock drift rate (sec/sec2) af2 (see *Br.Orbit-5*, data source, bits 8+9)	A1,I2.2,<br><br>1X,I4,<br>5(1X,I2.2),<br>3D19.12<br><br>*)
**BROADCAST ORBIT - 1**	- IODnav Issue of Data of the nav batch<br>- Crs (meters)<br>- Delta n (radians/sec)<br>- M0 (radians)	4X,4D19.12<br><br>***)
**BROADCAST ORBIT - 2**	- Cuc (radians)<br>- e Eccentricity<br>- Cus (radians)<br>- sqrt(a) (sqrt(m))	4X,4D19.12
**BROADCAST ORBIT - 3**	- Toe Time of Ephemeris (sec of GAL week)<br>- Cic (radians)<br>- OMEGA0 (radians)<br>- Cis (radians)	4X,4D19.12
**BROADCAST ORBIT - 4**	- i0 (radians)<br>- Crc (meters)<br>- omega (radians)<br>- OMEGA DOT (radians/sec)	4X,4D19.12
**BROADCAST ORBIT - 5**	- IDOT (radians/sec)<br>- Data sources (FLOAT --> INTEGER)<br>Bit 0 set: I/NAV E1-B<br>Bit 1 set: F/NAV E5a-I<br>Bit 2 set: I/NAV E5b-I<br>Bits 0-2 : non-exclusive<br>Bit 3 reserved for Galileo internal use<br>Bit 4 reserved for Galileo internal use<br>Bit 8 set: af0-af2, Toc, SISA are for E5a,E1<br>Bit 9 set: af0-af2, Toc, SISA are for E5b,E1<br>- GAL Week # (to go with Toe)<br>- spare	4X,4D19.12<br><br>****)
**BROADCAST ORBIT - 6**	- SISA Signal in space accuracy (meters)<br>Undefined/unknown: -1.0<br>- SV health (FLOAT converted to INTEGER)<br>Bit 0: E1B DVS<br>Bits 1-2: E1B HS	4X,4D19.12<br><br>*****)

TABLE A8 GNSS NAVIGATION MESSAGE FILE - GALILEO DATA RECORD DESCRIPTION		
**OBS. RECORD**	**DESCRIPTION**	**FORMAT**
	Bit 3: E5a DVS<br>Bits 4-5: E5a HS<br>Bit 6: E5b DVS<br>Bits 7-8: E5b HS<br>- BGD E5a/E1 (seconds)<br>- BGD E5b/E1 (seconds)	
**BROADCAST ORBIT - 7**	- Transmission time of message **)<br>(sec of GAL week, derived from WN and TOW of page type 1)<br>- spare<br>- spare<br>- spare	4X,4D19.12

*) In order to account for the various compilers, E,e,D, and d are allowed letters between the fraction and exponent of all floating point numbers in the navigation message files. Zero-padded two-digit exponents are required, however.

) Adjust the *Transmission time of message* by + or -604800 to refer to the reported week in ***BROADCAST ORBIT 5*, if necessary. Set value to 0.9999E9 if not known.

***) Angles and their derivatives transmitted in units of semi-circles and semi-circles/sec have to be converted to radians by the RINEX generator.

****) The GAL week number is a continuous number, aligned to (and hence identical to) the continuous GPS week number used in the RINEX navigation message files. The broadcast 12-bit Galileo System Time week has a roll-over after 4095. It started at zero at the first GPS roll-over (continuous GPS week 1024). Hence GAL week = GST week + 1024 + n*4096 (n: number of GST roll-overs).

*****)
-If bit 0 or bit 2 of Data sources (***BROADCAST ORBIT - 5)*** is set, `E1B DVS` & `HS`, `E5b DVS` & `HS` and both `BGD`s are valid. -If bit 1 of Data sources is set, `E5a DVS` & `HS` and `BGD E5a/E1` are valid. -Non valid parameters are set to 0 and to be ignored

A 9 GALILEO Navigation Message File – Example

```
+------------------------------------------------------------------------------+
|                                   TABLE A9                                   |
|                    GALILEO NAVIGATION MESSAGE FILE - EXAMPLE                 |
+------------------------------------------------------------------------------+

----|---1|0---|---2|0---|---3|0---|---4|0---|---5|0---|---6|0---|---7|0---|---8|

     3.02           N: GNSS NAV DATA    E: GALILEO          RINEX VERSION / TYPE
XXRINEXN V3         AIUB                20060902 192236 UTC PGM / RUN BY / DATE
EXAMPLE OF VERSION 3.02 FORMAT                              COMMENT

To be supplied later

----|---1|0---|---2|0---|---3|0---|---4|0---|---5|0---|---6|0---|---7|0---|---8|
```

A 10 GNSS Navigation Message File – GLONASS Data Record Description

TABLE A10<br>GNSS NAVIGATION MESSAGE FILE – GLONASS DATA RECORD DESCRIPTION		
**OBS. RECORD**	**DESCRIPTION**	**FORMAT**
SV / EPOCH / SV CLK	- Satellite system (**R**), satellite number (slot number in sat. constellation)<br>- Epoch: Toc - Time of Clock (UTC) year (4 digits)<br>- month, day, hour, minute, second<br>- SV clock bias (sec) (-TauN)<br>- SV relative frequency bias (+GammaN)<br>- Message frame time (tk+nd*86400) in seconds of the UTC week	A1,I2.2,<br><br>1X,I4,<br>5(1X,I2.2),<br>3D19.12<br><br>*)
**BROADCAST ORBIT - 1**	- Satellite position X (km)<br>- velocity X dot (km/sec)<br>- X acceleration (km/sec2)<br>- health (0=OK) (Bn)	4X,4D19.12
**BROADCAST ORBIT - 2**	- Satellite position Y (km)<br>- velocity Y dot (km/sec)<br>- Y acceleration (km/sec2)<br>- frequency number(-7...+13) (-7...+6 ICD 5.1)	4X,4D19.12
**BROADCAST ORBIT - 3**	- Satellite position Z (km)<br>- velocity Z dot (km/sec)<br>- Z acceleration (km/sec2)<br>- Age of oper. information (days) (E)	4X,4D19.12

*) In order to account for the various compilers, E,e,D, and d are allowed letters between the fraction and exponent of
all floating point numbers in the navigation message files. Zero-padded two-digit exponents are required, however.

A 11 GNSS Navigation Message File – Example: Mixed GPS / GLONASS

```
+------------------------------------------------------------------------------+
|                                  TABLE A11                                   |
|           GNSS NAVIGATION MESSAGE FILE – EXAMPLE MIXED GPS/GLONASS           |
+------------------------------------------------------------------------------+

----|---1|0---|---2|0---|---3|0---|---4|0---|---5|0---|---6|0---|---7|0---|---8|

     3.02           N: GNSS NAV DATA    M: MIXED            RINEX VERSION / TYPE
XXRINEXN V3         AIUB                20061002 000123 UTC PGM / RUN BY / DATE
EXAMPLE OF VERSION 3.02 FORMAT                              COMMENT
GPSA   0.1025E-07  0.7451E-08 -0.5960E-07 -0.5960E-07       IONOSPHERIC CORR
GPSB   0.8806E+05  0.0000E+00 -0.1966E+06 -0.6554E+05       IONOSPHERIC CORR
GPUT  0.2793967723E-08 0.000000000E+00 147456 1395          TIME SYSTEM CORR
GLUT  0.7823109626E-06 0.000000000E+00      0 1395          TIME SYSTEM CORR
    14                                                      LEAP SECONDS
                                                            END OF HEADER
G01 2006 10 01 00 00 00 0.798045657575E-04 0.227373675443E-11 0.000000000000E+00
     0.560000000000E+02-0.787500000000E+01 0.375658504827E-08 0.265129935612E+01
    -0.411644577980E-06 0.640150101390E-02 0.381097197533E-05 0.515371852875E+04
     0.000000000000E+00 0.782310962677E-07 0.188667086536E+00-0.391155481338E-07
     0.989010441512E+00 0.320093750000E+03-0.178449589759E+01-0.775925177541E-08
     0.828605943335E-10 0.000000000000E+00 0.139500000000E+04 0.000000000000E+00
     0.200000000000E+01 0.000000000000E+00-0.325962901115E-08 0.560000000000E+02
    -0.600000000000E+02 0.400000000000E+01
G02 2006 10 01 00 00 00 0.402340665460E-04 0.386535248253E-11 0.000000000000E+00
     0.135000000000E+03 0.467500000000E+02 0.478269921862E-08-0.238713891022E+01
     0.250712037086E-05 0.876975362189E-02 0.819191336632E-05 0.515372778320E+04
     0.000000000000E+00-0.260770320892E-07-0.195156738598E+01 0.128522515297E-06
     0.948630520258E+00 0.214312500000E+03 0.215165003775E+01-0.794140221985E-08
    -0.437875382124E-09 0.000000000000E+00 0.139500000000E+04 0.000000000000E+00
     0.200000000000E+01 0.000000000000E+00-0.172294676304E-07 0.391000000000E+03
    -0.600000000000E+02 0.400000000000E+01
R01 2006 10 01 00 15 00-0.137668102980E-04-0.454747350886E-11 0.900000000000E+02
     0.157594921875E+05-0.145566368103E+01 0.000000000000E+00 0.000000000000E+00
    -0.813711474609E+04 0.205006790161E+01 0.931322574615E-09 0.700000000000E+01
     0.183413398438E+05 0.215388488770E+01-0.186264514923E-08 0.100000000000E+01
R02 2006 10 01 00 15  0-0.506537035108E-04 0.181898940355E-11 0.300000000000E+02
     0.155536342773E+05-0.419384956360E+00 0.000000000000E+00 0.000000000000E+00
    -0.199011298828E+05 0.324192047119E+00-0.931322574615E-09 0.100000000000E+01
     0.355333544922E+04 0.352666091919E+01-0.186264514923E-08 0.100000000000E+01
----|---1|0---|---2|0---|---3|0---|---4|0---|---5|0---|---6|0---|---7|0---|---8|
```

A 12 GNSS Navigation Message File – QZSS Data Record Description

TABLE A12<br>QZSS NAVIGATION MESSAGE FILE – QZSS DATA RECORD DESCRIPTION		
**OBS. RECORD**<br>(Columns 61-80)	**DESCRIPTION**	**FORMAT**
**PRN / EPOCH / SV CLK**	- Satellite system (J), Satellite PRN-192<br>- Epoch: Toc - Time of Clock year (2 digits, padded with 0 if necessary)<br>month<br>day<br>hour<br>minutes<br>seconds<br>– SV clock bias (seconds)<br>– SV clock drift (sec/sec)<br>SV clock drift rate (sec/sec2)	A1,I2,<br><br>1X,I2.2,<br>1X,I2,<br>1X,I2,<br>1X,I2,<br>1X,I2,<br>F5.1,<br>3D19.12<br><br>*)
**BROADCAST ORBIT - 1**	– IODE Issue of Data, Ephemeris<br>– Crs (meters)<br>– Delta n (radians/sec)<br>– M0 (radians)	4X,4D19.12
**BROADCAST ORBIT - 2**	– Cuc (radians)<br>– e Eccentricity<br>– Cus (radians)<br>– sqrt(A) (sqrt(m))	4X,4D19.12
**BROADCAST ORBIT - 3**	– Toe Time of Ephemeris (sec of GPS week)<br>– Cic (radians)<br>– OMEGA (radians)<br>– CIS (radians)	4X,4D19.12
**BROADCAST ORBIT - 4**	– i0 (radians)<br>– Crc (meters)<br>– omega (radians)<br>– OMEGA DOT (radians/sec)	4X,4D19.12
**BROADCAST ORBIT – 5**	– IDOT (radians/sec)<br>– Codes on L2 channel (see IS-QZSS 5.2.2.2.3(2))<br>– GPS Week # (to go with TOE) Continuous number, not mod(1024)!<br>spare	4X,4D19.12
**BROADCAST ORBIT – 6**	– SV accuracy (meters) (see IS-GPS-200, 20.3.3.3.1.3)<br>– SV health (bits 17-22 w 3 sf 1) (see IS-QZSS 5.2.2.2.3(4))	4X,4D19.12

TABLE A12<br>QZSS NAVIGATION MESSAGE FILE – QZSS DATA RECORD DESCRIPTION		
**OBS. RECORD**<br>(Columns 61-80)	**DESCRIPTION**	**FORMAT**
	– TGD (seconds)<br>– IODC Issue of Data, Clock	
**BROADCAST ORBIT – 7**	- Transmission time of message ****)** (sec of GPS week, derived e.g. from Z-count in Hand Over Word (HOW)<br>- Fit interval (hours) (see IS-QZSS, 5.2.2.2.4(4)) Zero – one hour, 1 – more than 2 hours<br>- Spare<br>- Spare	4X,4D19.12

Records marked with * are optional

**)Adjust the Transmission time of message by -604800 to refer to the reported week, if necessary.

*) In order to account for the various compilers, letters E,e,D, and d are allowed between the fraction and exponent of all floating point numbers in the navigation message files. However, zero-padded two-digit exponents are required.

A 13 GNSS Navigation Message File – BDS Data Record Description

Table A13
GNSS NAVIGATION MESSAGE FILE – BDS DATA RECORD DESCRIPTION

OBS. RECORD	DESCRIPTION	FORMAT
SV /EPOCH / SV CLK	- Satellite system (C), sat number (PRN)<br>- Epoch: Toc - Time of Clock (BDT) year (4 digits)<br>- month, day, hour, minute, second<br>- SV clock bias (seconds)<br>- SV clock drift (sec/sec)<br>- SV clock drift rate (sec/sec^2)	A1,I2.2,<br>1X,I4<br><br>5,1X,I2.2,<br>3D19.12<br>*)
BROADCAST ORBIT – 1	- IODE Issue of Data, Ephemeris<br>- Crs (meters)<br>- Delta n (radians/sec)<br>- M0 (radians)	4X,4D19.12<br><br><br>**)
BROADCAST ORBIT – 2	- Cuc (radians)<br>- e Eccentricity<br>- Cus (radians)<br>- sqrt(A) (sqrt(m))	4X,4D19.12
BROADCAST ORBIT – 3	- Toe Time of Ephemeris (sec of BDT week)<br>- Cic (radians)<br>- OMEGA0 (radians)<br>- -Cis (radians)	4X,4D19.12
BROADCAST ORBIT – 4	- i0 (radians)<br>- Crc (meters)<br>- omega (radians)<br>- OMEGA DOT (radians/sec)	4X,4D19.12
BROADCAST ORBIT – 5	- IDOT (radians/sec)<br>- Spare<br>- BDT Week #<br>- Spare	4X,4D19.12<br><br>***)
BROADCAST ORBIT – 6	- SV accuracy (meters)<br>- SatH1<br>- TGD1 B1/B3 (seconds)<br>- TGD2 B2/B3 (seconds)	4X,4D19.12
BROADCAST ORBIT – 7	- Transmission time of message ****) (sec of BDT week,)<br>- IODC Issue of Data Clock<br>- Spare<br>- Spare	4X,4D19.12
*) In order to account for the various compilers, E,e,D, and d are allowed letters between the fraction and exponent of all floating point numbers in the navigation message files. Zero-padded two-digit exponents		

are required, however.

**) Angles and their derivatives transmitted in units of semi-circles and semi-circles/sec have to be converted to radi¬ans by the RINEX generator.

***) The BDT week number is a continuous number. The broadcast 13-bit BDS System Time week has a roll-over after 8191. It started at zero at 1-Jan-2006, Hence BDT week = BDT week_BRD + (n*8192) where (n: number of BDT roll-overs).

****)Adjust the Transmission time of message by + or -604800 to refer to the reported week in BROADCAST ORBIT -5, if necessary. Set value to 0.9999E9 if not known.

A 14 GNSS Navigation Message File – SBAS Data Record Description

TABLE A14<br>GNSS NAVIGATION MESSAGE FILE – SBAS/QZSS L1 SAIF DATA RECORD DESCRIPTION		
**OBS. RECORD**	**DESCRIPTION**	**FORMAT**
**SV / EPOCH / SV CLK**	- Satellite system (**S**), satellite number (slot number in sat. constellation)<br>- Epoch: Toc - Time of Clock (GPS) year (4 digits)<br>- month, day, hour, minute, second<br>- SV clock bias (sec) (aGf0)<br>- SV relative frequency bias (aGf1)<br>- Transmission time of message (start of the message) in GPS seconds of the week	A1,I2.2,<br><br>1X,I4,<br>5(1X,I2.2),<br>3D19.12,<br><br>*)
**BROADCAST ORBIT - 1**	- Satellite position X (km)<br>- velocity X dot (km/sec)<br>- X acceleration (km/sec2)<br>- health (0=OK)	4X,4D19.12
**BROADCAST ORBIT - 2**	- Satellite position Y (km)<br>- velocity Y dot (km/sec)<br>- Y acceleration (km/sec2)<br>- Accuracy code (URA, meters)	4X,4D19.12
**BROADCAST ORBIT - 3**	- Satellite position Z (km)<br>- velocity Z dot (km/sec)<br>- Z acceleration (km/sec2)<br>- IODN (Issue of Data Navigation, DO229, 8 first bits after Message Type if MT9)	4X,4D19.12

*) In order to account for the various compilers, E,e,D, and d are allowed letters between the fraction and exponent of all floating point numbers in the navigation message files. Zero-padded two-digit exponents are required, however.

For QZSS L1-SAIF, note that accelerations represent only lunar and solar perturbation terms and satellite position can be computed based on equations in Section A.3.1.2 of GLONASS ICD version 5.0.

A 15 SBAS Navigation Message File -Example

```
+------------------------------------------------------------------------------+
|                              TABLE A15                                       |
|                SBAS NAVIGATION MESSAGE FILE - EXAMPLE                        |
+------------------------------------------------------------------------------+
----|---1|0---|---2|0---|---3|0---|---4|0---|---5|0---|---6|0---|---7|0---|---8|

     3.02           N: GNSS NAV DATA    S: SBAS             RINEX VERSION / TYPE
SBAS2RINEX 3.0      CNES                20031018 140100     PGM / RUN BY / DATE
EXAMPLE OF VERSION 3.02 FORMAT                              COMMENT
SBUT  -.1331791282D-06 -.107469589D-12 552960 1025 EGNOS  5 TIME SYSTEM CORR
    13                                                      LEAP SECONDS
This file contains navigation message data from a SBAS      COMMENT
(geostationary) satellite, here AOR-W (PRN 122 = # S22)     COMMENT
                                                            END OF HEADER
S22 2003 10 18  0  1  4-1.005828380585D-07 6.366462912410D-12 5.184420000000D+05
     2.482832392000D+04-3.593750000000D-04-1.375000000000D-07 0.000000000000D+00
    -3.408920872000D+04-1.480625000000D-03-5.000000000000D-08 4.000000000000D+00
    -1.650560000000D+01 8.360000000000D-04 6.250000000000D-08 2.300000000000D+01
S22 2003 10 18  0  5 20-9.872019290924D-08 5.456968210638D-12 5.186940000000D+05
     2.482822744000D+04-3.962500000000D-04-1.375000000000D-07 0.000000000000D+00
    -3.408958936000D+04-1.492500000000D-03-5.000000000000D-08 4.000000000000D+00
    -1.628960000000D+01 8.520000000000D-04 6.250000000000D-08 2.400000000000D+01
S22 2003 10 18  0  9 36-9.732320904732D-08 4.547473508865D-12 5.189510000000D+05
     2.482812152000D+04-4.325000000000D-04-1.375000000000D-07 0.000000000000D+00
    -3.408997304000D+04-1.505000000000D-03-5.000000000000D-08 4.000000000000D+00
    -1.606960000000D+01 8.800000000000D-04 6.250000000000D-08 2.500000000000D+01
S22 2003 10 18  0 13 52-9.592622518539D-08 4.547473508865D-12 5.192110000000D+05
     2.482800632000D+04-4.681250000000D-04-1.375000000000D-07 0.000000000000D+00
    -3.409035992000D+04-1.518125000000D-03-3.750000000000D-08 4.000000000000D+00
    -1.584240000000D+01 8.960000000000D-04 6.250000000000D-08 2.600000000000D+01

----|---1|0---|---2|0---|---3|0---|---4|0---|---5|0---|---6|0---|---7|0---|---8|
```

A 16 Meteorological Data File -Header Section Description

TABLE A16<br>METEOROLOGICAL DATA FILE - HEADER SECTION DESCRIPTION		
**HEADER LABEL**<br>(Columns 61-80)	**DESCRIPTION**	**FORMAT**
**RINEX VERSION / TYPE**	- Format version : **3.02**<br>- File type: **M** for Meteorological Data	F9.2,11X,<br>A1,39X
**PGM / RUN BY / DATE**	- Name of program creating current file<br>- Name of agency creating current file<br>- Date of file creation (See section 5.8)	A20,<br>A20,<br>A20
*** COMMENT**	- Comment line(s)	A60
**MARKER NAME**	- Station Name (preferably identical to **MARKER NAME** in the associated Observation File)	A60
*** MARKER NUMBER**	- Station Number (preferably identical to **MARKER NUMBER** in the associated Observation File)	A20
**# / TYPES OF OBSERV**	- Number of different observation types stored in the file<br>- Observation types<br>- The following meteorological observation types are defined in RINEX Version 3:<br>- **PR** : Pressure (mbar)<br>- **TD** : Dry temperature (deg Celsius)<br>- **HR** : Relative humidity (percent)<br>- **ZW** : Wet zenith path delay (mm), (for WVR data)<br>- **ZD** : Dry component of zen.path delay (mm)<br>- **ZT** : Total zenith path delay (mm)<br>- **WD** : Wind azimuth (deg) from where the wind blows<br>- **WS** : Wind speed (m/s)<br>- **RI** : "Rain increment" (1/10 mm): Rain accumulation since last measurement<br>- **HI** : Hail indicator non-zero: Hail detected since last measurement<br>- The sequence of the types in this record must correspond to the sequence of the measurements in the data records.<br>- If more than 9 observation types are being used, use continuation lines with format (6X,9(4X,A2))	I6,<br><br>9(4X,A2)

TABLE A16<br>METEOROLOGICAL DATA FILE - HEADER SECTION DESCRIPTION		
**HEADER LABEL**<br>(Columns 61-80)	**DESCRIPTION**	**FORMAT**
**SENSOR MOD/TYPE/ACC**	Description of the met sensor<br>- Model (manufacturer)<br>- Type<br>- Accuracy (same units as obs values)<br>- Observation type<br>Record is repeated for each observation type found in **# / TYPES OF OBSERV** record	 A20,<br>A20,6X,<br>F7.1,4X,<br>A2,1X
**SENSOR POS XYZ/H**	- Approximate position of the met sensor -<br>Geocentric coordinates X,Y,Z (ITRF<br>- Ellipsoidal height H or WGS-84)<br>- Observation type<br>Set X,Y,Z to zero if not known.<br>Make sure H refers to ITRF or WGS-84!<br>Record required for barometer, recommended for other sensors.	 3F14.4,<br>1F14.4,<br>1X,A2,1X
**END OF HEADER**	Last record in the header section.	60X

Records marked with * are optional

A 17 Meteorological Data File -Data Record Description

TABLE A17<br>METEOROLOGICAL DATA FILE - DATA RECORD DESCRIPTION		
**OBS. RECORD**	**DESCRIPTION**	**FORMAT**
**EPOCH / MET**	- Epoch in GPS time (not local time!) year (4 digits, padded with 0 if necessary)<br>- month, day, hour, min, sec<br>- Met data in the same sequence as given in the header<br>More than 8 met data types: Use continuation lines	1X,I4.4,<br>5(1X,I2),<br>mF7.1<br>4X,10F7.1

A 18 Meteorological Data File – Example

```
+----------------------------------------------------------------------------+
|                                TABLE A18                                   |
|                    METEOROLOGICAL DATA FILE - EXAMPLE                      |
+----------------------------------------------------------------------------+

----|---1|0---|---2|0---|---3|0---|---4|0---|---5|0---|---6|0---|---7|0---|---8|

     3.02           METEOROLOGICAL DATA                     RINEX VERSION / TYPE
XXRINEXM V9.9       AIUB                19960401 144333 UTC PGM / RUN BY / DATE
EXAMPLE OF A MET DATA FILE                                  COMMENT
A 9080                                                      MARKER NAME
     3    PR    TD    HR                                    # / TYPES OF OBSERV
PAROSCIENTIFIC      740-16B                       0.2    PR SENSOR MOD/TYPE/ACC
HAENNI                                            0.1    TD SENSOR MOD/TYPE/ACC
ROTRONIC            I-240W                        5.0    HR SENSOR MOD/TYPE/ACC
        0.0000        0.0000        0.0000        1234.5678 PR SENSOR POS XYZ/H
                                                            END OF HEADER
 1996  4  1  0  0 15  987.1   10.6   89.5
 1996  4  1  0  0 30  987.2   10.9   90.0
 1996  4  1  0  0 45  987.1   11.6   89.0

----|---1|0---|---2|0---|---3|0---|---4|0---|---5|0---|---6|0---|---7|0---|---8|
```

A 19 Reference Code and Phase Alignment by Constellation and Frequency Band

TABLE A19
Reference Code and Phase Alignment by Frequency Band

System	Frequency Band	Frequency [MHz]	Signal	RINEX Observation Code	Phase Correction applied to each observed phase to obtain aligned phase. (φRINEX = φ original(as issued by the SV) + Δφ)
GPS	L1	1575.42	C/A	L1C	None (Reference Signal)
			L1C-D	L1S	+¼ cycle
			L1C-P	L1L	+¼ cycle
			L1C-(D+P)	L1X	+¼ cycle
			P	L1P	+¼ cycle
			Z-tracking	L1W	+¼ cycle
			Codeless	L1N	+¼ cycle
	L2 See Note 1	1227.60	C/A	L2C	For Block II/IIA/IIR – None; For Block IIR-M/IIF/III -¼ cycle See Note 2
			Semi-codeless	L2D	None
			L2C(M)	L2S	-¼ cycle
			L2C(L)	L2L	-¼ cycle
			L2C(M+L)	L2X	-¼ cycle
			P	L2P	None (Reference Signal)
			Z-tracking	L2W	None
			Codeless	L2N	None
	L5	1176.45	I	L5I	None (Reference Signal)
			Q	L5Q	-¼ cycle
			I+Q	L5X	Must be aligned to L5I
GLONASS	G1	1602+k*9/16	C/A	L1C	None (Reference Signal)
			P	L1P	+¼ cycle
	G2	1246+k*7/16	C/A	L2C	None (Reference

TABLE A19<br>Reference Code and Phase Alignment by Frequency Band					
**System**	**Frequency Band**	**Frequency [MHz]**	**Signal**	**RINEX Observation Code**	**Phase Correction applied to each observed phase to obtain aligned phase.**<br><br>**(φRINEX = φ original(as issued by the SV) + Δφ)**
					Signal)
			P	L2P	+¼ cycle
	G3	1202.025	I	L3I	None (Reference Signal)
			Q	L3Q	-¼ cycle
			I+Q	L3X	Must be aligned to L3I
Galileo	E1	1575.42	B I/NAV OS/CS/SoL	L1B	None (Reference Signal)
			C no data	L1C	+½ cycle
			B+C	L1X	Must be aligned to L1B
	E5A	1176.45	I	L5I	None(Reference Signal)
			Q	L5Q	-¼ cycle
			I+Q	L5X	Must be aligned to L5I
	E5B	1207.140	I	L7I	None (Reference Signal)
			Q	L7Q	-¼ cycle
			I+Q	L7X	Must be aligned to L7I
	E5(A+B)	1191.795	I	L8I	None (Reference Signal)
			Q	L8Q	-¼ cycle
			I+Q	L8X	Must be aligned to L8I
	E6	1278.75	B	L6B	None (Reference Signal)
			C	L6C	-½ cycle
			B+C	L6X	Must be aligned to L6B
QZSS	L1	1575.42	C/A	L1C	None (Reference Signal)
			L1C (D)	L1S	None
			L1C (P)	L1L	+¼ cycle
			L1C-(D+P)	L1X	+¼ cycle
			L1-SAIF	L1Z	N/A
	L2	1227.60	L2C (M)	L2S	None (Reference

**TABLE A19<br>Reference Code and Phase Alignment by Frequency Band**					
**System**	**Frequency Band**	**Frequency [MHz]**	**Signal**	**RINEX Observation Code**	**Phase Correction applied to each observed phase to obtain aligned phase.**<br><br>**(φRINEX = φ original(as issued by the SV) + Δφ)**
					Signal)
			L2C (L)	L2L	None
			L2C (M+L)	L2X	None
	L5	1176.45	I	L5I	None (Reference Signal)
			Q	L5Q	-¼ cycle
			I+Q	L5X	Must be aligned to L5I
	LEX(6)	1278.75	S	L6S	None (Reference Signal)
			L	L6L	None
			S+L	L6X	None
BDS	B1	1561.098	I	L1I	None (Reference Signal)
			Q	L1Q	-¼ cycle
			I+Q	L1X	Must be aligned to L2I
	B2	1207.140	I	L7I	None (Reference Signal)
			Q	L7Q	-¼ cycle
			I+Q	L7X	Must be aligned to L7I
	B3	1268.52	I	L6I	None (Reference Signal)
			Q	L6Q	-¼ cycle
			I+Q	L6X	Must be aligned to L6I

NOTES:

1) The GPS L2 phase shift values ignore FlexPower when the phases of the L2E and L2C can be changed on the satellite.

2) The phase of the L2 C/A signal is dependent on the GPS satellite generation.
