# Supplementary material for: Improved GNSS integer ambiguity resolution method based on the column oriented Cholesky decomposition
Source: Sci Rep. 2023 Mar 17;13:4454. doi: 10.1038/s41598-023-31635-3 (PMC10023790; doi:10.1038/s41598-023-31635-3)
Supplement: Supplementary file 1 — Supplementary Information. [file 41598_2023_31635_MOESM1_ESM.zip › supplement materials of the manuscript/Figure 6/Figure 6 Data Description.doc]

- Measured experiment/Figure 6（a）-hksl2000.21o is the observation file of long baseline；

Measured experiment/Figure 6（a）-jfng2000.21o is the observation file of long baseline；

Measured experiment/Figure 6（a）-brdm2000.21p is the satellite files of long baseline,

the corresponding results of this data are shown in Figure 6（a）。

- Measured experiment/Figure 6（b）-hksl2000.21o is the observation file of long baseline；

Measured experiment/Figure 6（b）-jfng2000.21o is the observation file of long baseline；

Measured experiment/Figure 6（b）-brdm2000.21p is the satellite files of long baseline,

the corresponding results of this data are shown in Figure 6（b）。

- Measured experiment/Figure 6（c）-hksl2000.21o is the observation file of long baseline；

Measured experiment/Figure 6（c）-jfng2000.21o is the observation file of long baseline；

Measured experiment/Figure 6（c）-brdm2000.21p is the satellite files of long baseline,

- Measured experiment/the corresponding results of this data are shown in Figure 6（c）。Short baseline solution time.xlsx Medium baseline solution time.xlsx and Long baseline solution time.xlsx are respectively the calculation time of the two methods of baseline and long baseline in the short baseline. The first column is the calculation time of Choleksy algorithm, and the second column is the calculation time of C-Choleksy algorithm. The data in Figure 6 is obtained from Short baseline solution time.xlsx Medium baseline solution time.xlsx and Long baseline solution time.xlsx by drawing CDF diagram in matlab。
- All measured experiment data conform to GNSS standard data format, i.e. RINEX format. Format description is as follows rinex302. pdf
